# Supplementary material for: Native-to-invasive rodent species turn-over within African cities: The example of Niamey, Niger
Source: PLoS One. 2025 Jul 22;20(7):e0325427. doi: 10.1371/journal.pone.0325427 (PMC12282928; doi:10.1371/journal.pone.0325427)
Supplement: S1 Table — Data are organized by trap type and locality. The term “Stats” refers to the use of Holm-adjusted two-tailed Wilcoxon test p-values. Where possible (i.e., for localities where at least one individual was captured), capture rates by trap and species were also compared at locality level using the same procedure. (DOCX) [file pone.0325427.s001.docx]

|  |  | **Wire mesh trap** | **Sherman trap** |
| --- | --- | --- | --- |
|  |  | ***Stats*** | ***Stats*** |
| ***Species*** | **Locality** |  |  |
| *Rattus rattus* | BOU | p=0.00031 | p=0.41 |
|  | CYA | p=0.0075 | p=0.53 |
|  | DAR | p=0.0054 | p=0.53 |
|  | GAM | p=0.39 | p=0.39 |
|  | GRM | p=0.036 | p=0.85 |
|  | KAR | p<0.001 | NA |
|  | WAD | p=0.053 | p=0.14 |
|  | subtotal | p=1.3e-9 | p=0.049 |
| *Mastomys nataleis* | BOU | p=0.13 | p=0.0028 |
|  | CYA | p=0.47 | p=3e-4 |
|  | DAR | p=0.14 | p=0.00048 |
|  | GAM | p=0.73 | p=0.18 |
|  | KAR | p=0.95 | p=0.0013 |
|  | KOT | p=0.01 | p=0.1 |
|  | TCH | p=0.17 | p=0.79 |
|  | WAD | p=0.35 | p=0.0066 |
|  | subtotal | p=0.22 | p=9.7e-6 |
| *Crocidura olivieri* | BOU | p=0.045 | p=0.93 |
|  | CYA | p=0.34 | NA |
|  | DAR | p=0.11 | NA |
|  | GRM | NA | P=0.1 |
|  | KAR | p=0.4 | p=0.035 |
|  | KOT | p=0.42 | NA |
|  | TCH | NA | P=0.3 |
|  | WAD | NA | P=0.21 |
|  | subtotal | p=0.085 | p=0.0046 |
| *Mus musculus* | GRM | p=0.14 | p~1 |

**Table S1.** **Results of statistical tests of capture rates comparisons between periods.**

Data are organized by trap type and locality. The term “Stats” refers to the use of Holm-adjusted two-tailed Wilcoxon test p-values. Where possible (i.e. for localities where at least one individual was captured), capture rates by trap and species were also compared at locality level using the same procedure.
