# Supplementary figures and images for: Native-to-invasive rodent species turn-over within African cities: The example of Niamey, Niger
Source: PLoS One. 2025 Jul 22;20(7):e0325427. doi: 10.1371/journal.pone.0325427 (PMC12282928; doi:10.1371/journal.pone.0325427)

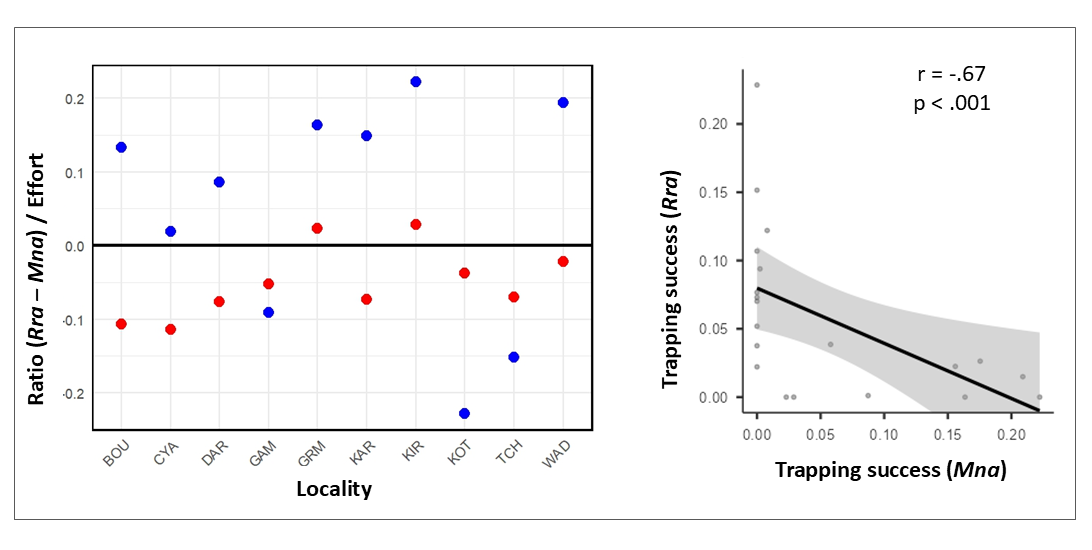

Supplement: S1 Fig — The panel on the right shows the evolution of the ratio [(R. rattus – M. natalensis)/Nb of trap-nights] between the two periods. The red dots indicate the ratios of period 1 while the blues indicate those of period 2. the line at 0 separates the positives (above) from the negatives (below) values. The left-panel shows the Spearman correlation graph between the trapping success of the two species. (TIF) [file pone.0325427.s002.tif]
