# Supplementary material for: Native-to-invasive rodent species turn-over within African cities: The example of Niamey, Niger
Source: PLoS One. 2025 Jul 22;20(7):e0325427. doi: 10.1371/journal.pone.0325427 (PMC12282928; doi:10.1371/journal.pone.0325427)
Supplement: Attachment — Submitted filename: PONE-D-24-55809_reviewer.pdf [file pone.0325427.s003.pdf]

PLOS ONE

# Native-to-invasive rodent species turn-over within African cities: the example of Niamey, Niger

--Manuscript Draft--

| | |
|---|---|
| **Manuscript Number:** | PONE-D-24-55809 |
| **Article Type:** | Research Article |
| **Full Title:** | Native-to-invasive rodent species turn-over within African cities: the example of Niamey, Niger |
| **Short Title:** | Native-to-invasive rodent species turn-over within African cities: the example of Niamey, Niger |
| **Corresponding Author:** | Abdoulaziz Ibrahim Danzabarma<br>Université Abdou Moumouni de Niamey Faculté des Sciences et Techniques: Universite Abdou Moumouni de Niamey Faculte des Sciences et Technique<br>Niamey, NIGER |
| **Keywords:** | Biological invasion, urbanization, community ecology, zoonoses, rodents, Africa |
| **Abstract:** | Expanding transportation infrastructure has facilitated the introduction and proliferation of invasive small mammals, particularly now cosmopolitan rodents like Rattus spp. and Mus musculus, within urban socio-ecosystems where they may severely impact local biodiversity, economy and public health. However, fine-scale dynamics of such biological invasions are still poorly documented, especially in African cities where such biological invasions are still ongoing. We took advantage of a long-term monitoring program of terrestrial small mammal communities of Niamey, the capital city of Niger, to compare rodent and shrew faunas at the same sampling points ten years apart, namely in 2009-2013 and in 2020-2023. We show that R. rattus has been expanding significantly in almost all trapping areas across the city, while Mastomys natalensis relative abundance has decreased. This clear trend of native-to-invasive rodent species turnover was particularly marked within the core city, but some notable exceptions exist in peripheral as well as traditionally built zones where the native species still remains dominant. These patterns are discussed, with special attention given to possible human socio-economic and health implications. |
| **Order of Authors:** | Abdoulaziz Ibrahim Danzabarma |
| | Karmadine Hima |
| | Madougou Garba |
| | Seydou Issaka |
| | Gauthier Dobigny |
| **Additional Information:** | |
| **Question** | **Response** |
| **Financial Disclosure**<br><br>Enter a financial disclosure statement that describes the sources of funding for the work included in this submission. Review the submission guidelines for detailed requirements. View published research articles from *PLOS ONE* for specific examples.<br><br>This statement is required for submission and **will appear in the published article** if | Yes |

*Powered by Editorial Manager® and ProduXion Manager® from Aries Systems Corporation*

| | |
|---|---|
| the submission is accepted. Please make sure it is accurate.<br><br>**Funded studies**<br>Enter a statement with the following details:<br>• Initials of the authors who received each award<br>• Grant numbers awarded to each author<br>• The full name of each funder<br>• URL of each funder website<br>• Did the sponsors or funders play any role in the study design, data collection and analysis, decision to publish, or preparation of the manuscript?<br><br>Did you receive funding for this work? | |
| Please add funding details.<br>as follow-up to "**Financial Disclosure**<br><br>Enter a financial disclosure statement that describes the sources of funding for the work included in this submission. Review the submission guidelines for detailed requirements. View published research articles from *PLOS ONE* for specific examples.<br><br>This statement is required for submission and **will appear in the published article** if the submission is accepted. Please make sure it is accurate.<br><br>**Funded studies**<br>Enter a statement with the following details:<br>• Initials of the authors who received each award<br>• Grant numbers awarded to each author<br>• The full name of each funder<br>• URL of each funder website<br>• Did the sponsors or funders play any role in the study design, data collection and analysis, decision to publish, or preparation of the manuscript?<br><br>Did you receive funding for this work?" | GD, SCARIA Project funded by Belmont Forum<br>AID, Phd Thesis scholarship funded by IRD |
| Please select the country of your main | FRANCE - FR |

| | |
|---|---|
| research funder (please select carefully as in some cases this is used in fee calculation).<br>as follow-up to "**Financial Disclosure**<br><br>Enter a financial disclosure statement that describes the sources of funding for the work included in this submission. Review the submission guidelines for detailed requirements. View published research articles from *PLOS ONE* for specific examples.<br><br>This statement is required for submission and **will appear in the published article** if the submission is accepted. Please make sure it is accurate.<br><br>**Funded studies**<br>Enter a statement with the following details:<br>• Initials of the authors who received each award<br>• Grant numbers awarded to each author<br>• The full name of each funder<br>• URL of each funder website<br>• Did the sponsors or funders play any role in the study design, data collection and analysis, decision to publish, or preparation of the manuscript?<br><br>Did you receive funding for this work?" | |
| **Competing Interests**<br><br>Use the instructions below to enter a competing interest statement for this submission. On behalf of all authors, disclose any competing interests that could be perceived to bias this work—acknowledging all financial support and any other relevant financial or non-financial competing interests.<br><br>This statement is required for submission and **will appear in the published article** if the submission is accepted. Please make sure it is accurate and that any funding sources listed in your Funding Information | The authors have declared that no competing interests exist. |

| | |
|---|---|
| later in the submission form are also declared in your Financial Disclosure statement.<br><br>View published research articles from *PLOS ONE* for specific examples.<br><br>**NO authors have competing interests**<br><br>Enter: *The authors have declared that no competing interests exist.*<br><br>**Authors with competing interests**<br><br>Enter competing interest details beginning with this statement:<br><br>*I have read the journal's policy and the authors of this manuscript have the following competing interests: [insert competing interests here]*<br><br>* typeset | |
| **Ethics Statement**<br><br>Enter an ethics statement for this submission. This statement is required if the study involved:<br><br>• Human participants<br>• Human specimens or tissue<br>• Vertebrate animals or cephalopods<br>• Vertebrate embryos or tissues<br>• Field research<br><br>Write "N/A" if the submission does not require an ethics statement.<br><br>General guidance is provided below. Consult the submission guidelines for detailed instructions. **Make sure that all information entered here is included in the Methods section of the manuscript.** | Field permit number: N°054/2022/CNERS<br>Name of the institution: Ministry for Public Health of Niger Republic |

| | |
|---|---|
| **Format for specific study types**<br><br>**Human Subject Research (involving human participants and/or tissue)**<br>• Give the name of the institutional review board or ethics committee that approved the study<br>• Include the approval number and/or a statement indicating approval of this research<br>• Indicate the form of consent obtained (written/oral) or the reason that consent was not obtained (e.g. the data were analyzed anonymously)<br><br>**Animal Research (involving vertebrate animals, embryos or tissues)**<br>• Provide the name of the Institutional Animal Care and Use Committee (IACUC) or other relevant ethics board that reviewed the study protocol, and indicate whether they approved this research or granted a formal waiver of ethical approval<br>• Include an approval number if one was obtained<br>• If the study involved *non-human primates*, add *additional details* about animal welfare and steps taken to ameliorate suffering<br>• If anesthesia, euthanasia, or any kind of animal sacrifice is part of the study, include briefly which substances and/or methods were applied<br><br>**Field Research**<br><br>Include the following details if this study involves the collection of plant, animal, or other materials from a natural setting:<br>• Field permit number<br>• Name of the institution or relevant body that granted permission | |
| **Data Availability**<br><br>Authors are required to make all data underlying the findings described fully available, without restriction, and from the time of publication. PLOS allows rare exceptions to address legal and ethical concerns. See the PLOS Data Policy and FAQ for detailed information. | Yes - all data are fully available without restriction |

| | |
|---|---|
| A Data Availability Statement describing where the data can be found is required at submission. Your answers to this question constitute the Data Availability Statement and **will be published in the article**, if accepted.<br><br>**Important:** Stating 'data available on request from the author' is not sufficient. If your data are only available upon request, select 'No' for the first question and explain your exceptional situation in the text box.<br><br>Do the authors confirm that all data underlying the findings described in their manuscript are fully available without restriction? | |
| **Describe where the data may be found in full sentences. If you are copying our sample text, replace any instances of XXX with the appropriate details.**<br><br>• If the data are **held or will be held in a public repository**, include URLs, accession numbers or DOIs. If this information will only be available after acceptance, indicate this by ticking the box below. For example: *All XXX files are available from the XXX database (accession number(s) XXX, XXX.).*<br>• If the data are all contained **within the manuscript and/or Supporting Information files**, enter the following: *All relevant data are within the manuscript and its Supporting Information files.*<br>• If neither of these applies but you are able to provide **details of access elsewhere**, with or without limitations, please do so. For example:<br><br>*Data cannot be shared publicly because of [XXX]. Data are available from the XXX Institutional Data Access / Ethics Committee (contact via XXX) for researchers who meet the criteria for access to confidential data.*<br><br>*The data underlying the results presented in the study are available from (include the name of the third party* | All relevant data are within the manuscript and its Supporting Information files. |

| | |
|---|---|
| *and contact information or URL).*<br>• This text is appropriate if the data are owned by a third party and authors do not have permission to share the data.<br><br>* typeset | |
| Additional data availability information: | Tick here if your circumstances are not covered by the questions above and you need the journal's help to make your data available. |

Powered by Editorial Manager® and ProduXion Manager® from Aries Systems Corporation

Click here to access/download;Manuscript;Ibrahim et al_2024_Turn over.docx

# Native-to-invasive rodent species turn-over within African cities: the example of Niamey, Niger

Abdoulaziz Ibrahim Danzabarma [1,3*], Karmadine Hima[1], Madougou Garba [2], Seydou Issaka[1] & Gauthier Dobigny[3,4]

[1] Université Abdou Moumouni, Département de Biologie Animale, Niamey, Niger.

[2] Direction Générale de la Protection des Végétaux, Niamey, Niger.

[3] Institut de Recherche pour le Développement, Centre de Biologie pour la Gestion des Populations, Montferrier-sur-Lez, France.

[4] Institut Pasteur de Madagascar, Unité Peste, Antananarivo, Madagascar.

* <az.danzabarma@gmail.com>

# Abstract

Expanding transportation infrastructure has facilitated the introduction and proliferation of invasive small mammals, particularly now cosmopolitan rodents like *Rattus spp*. and *Mus musculus*, within urban socio-ecosystems where they may severely impact local biodiversity, economy and public health. However, fine-scale dynamics of such biological invasions are still poorly documented, especially in African cities where such biological invasions are still ongoing. We took advantage of a long-term monitoring program of terrestrial small mammal communities of Niamey, the capital city of Niger, to compare rodent and shrew faunas at the same sampling points ten years apart, namely in 2009-2013 and in 2020-2023. We show that *R. rattus* has been expanding significantly in almost all trapping areas across the city, while *Mastomys natalensis* relative abundance has decreased. This clear trend of native-to-invasive rodent species turnover was particularly marked within the core city, but some notable exceptions exist in peripheral as well as traditionally built zones where the native species still remains dominant. These patterns are discussed, with special attention given to possible human socio-economic and health implications.

# 1. Introduction

Rodents represent the most diverse group of mammals, exhibiting a worldwide distribution across a wide range of environments on all continents [1,2]. Some species are highly adaptable, a trait that has enabled them to colonize a diverse panel of terrestrial habitats, even those that have been deeply human-modified [3,4]. In particular, a few cosmopolitan invasive species, such as rats and mice, have been dominating small mammal communities in many urban areas

worldwide [5,6] where they may have highly deleterious impacts on native species assemblages [7,8] as well as human societies through socio-economic burden [9] and public health issues [10,11].

However, Africa may represent an exception where rats and mice are currently still in the process of inland dissemination across human settlement networks, often through road and/or fluvial transports [12–19]. The displacement of the highly prolific and native rodent species, especially *Mastomys spp*., by invasive black rats or house mice have been well documented in Senegal [14,15,17,19]. It is also apparent on the basis of ancient vs. more recent studies of rodents in some African cities. For instance, monitoring of small mammals in Dakar, Senegal, in the late 1920s [20] and 2016 [21] unambiguously show that native species were almost completely replaced by invasive ones, essentially house mice. Actually, available results from large city-scale trapping campaigns in Africa suggest that 99%, 96.8% and 65% of small terrestrial mammals (i.e., rodents and shrews) from Durban (South Africa), Makurdi (Nigeria) and Cotonou (Benin) belong to invasive species, namely house mice, black and/or Norway rats. [22–24].

Niger is a semi-arid to arid landlocked West African country. The capital city, Niamey, lies on both sides of the Niger River. Major tarred roads link the city to Benin southward, Mali northward, Burkina-Faso westward, and to the rest of the country as well as Nigeria eastward. Extensive studies conducted between 2009 and 2013 on small mammals all over the city have shown that the rodent community was largely dominated by the native species *M. natalensis* which was widespread and abundant in most households [16]. However, the invasive species *Rattus rattus* and *Mus musculus* were found in a few restricted areas of the core city, essentially corresponding to industrial sites (black rats), markets and their surroundings (both species)

where they were hypothetized to displace local rodents [16]. In addition, investigations based on large-scale rodent census, interviews and population genetics data [8,13] suggested that black rats and mice had been rather recently introduced, that invasion processes may still be ongoing, resulting from long-distance transports potentially from multiple geographic origins. If true, one expects that rats and mice may constitute important game changers, inducing drastic changes in small mammal community structure within Niamey with potential socio-economic and health implications.

In order to further document the dynamics and effects of this apparently ongoing rodent invasion process, we organized new small mammal monitoring campaigns between 2020 and 2023, notably targeting urban zones that had been sampled ten years before, and we compared them with the 2009-2013 results [16]. Doing so, we show a clear trend of black rats' spatial expansion in many areas of Niamey and we confirm that the species indeed tends to displace *M. natalensis*. As such, our study captures an unambiguous native-to-invasive rodent species turnover in an African city within a ten-year long period.

# 2. Materials and Methods

## 2.1. Data available from 2009-2013 rodent trapping campaigns

In the 2009-2013 study (hereafter designed as to period P1; [16]), the term "localities" refers to neighborhoods of Niamey; within each locality, they investigated specific places referred to as "sites" where trapping was carried out: these included households, industrial complexes and or market gardens. Here we follow their terminology in order to facilitate comparisons

between the two studies. In their study, 26 localities, including 18 neighborhoods, were sampled with an effort of 4 to 18 sites per locality, 7 market gardens as well as 2 industrial areas.

## 2.2. New rodent trapping campaigns

Small mammal assemblages within human settlements have been extensively studied using morphological, cytogenetic, DNA sequencing and genotyping approaches in Niger in general, and in Niamey in particular (e.g., [13,25]), including those from the P1 campaigns [16]. As a consequence, diversity within the city is now very well described, allowing us to rely confidently on morphological characteristics as well as external measurements (weight, body and tail length, ear length and hindfoot length) as described by Granjon and Duplantier [26] to identify the specimens collected during the sampling campaign newly presented here.

During the 2020-2023 period (hereafter designed as to period P2), 21 localities were sampled (Tab. 1), including 13 ones that had already been investigated during P1 (see localities in bold in Tab.1). For the latter neighborhoods monitored during both P1 and P2 campaigns, each time this was feasible, the same sites were re-sampled. Each session consisted in three consecutive nights of trapping in one given locality. Two models of traps, namely Sherman© (here after designed as to "Sh"; 8x9x23 cm; H.B. Sherman Traps, Inc., Tallahassee, FL, USA) and locally manufactured wire mesh ("Wm"; 10x10x25 cm) traps were used since they were shown to show significantly different success rate depending on the species, with Wm being more successful at capturing *Rattus spp.* while Sh were more performant for *M. natalensis*, *Mus musculus* and the native shrew *Crocidura olivieri* [16,27,28]. The baits were made of peanut butter and smoked fish or sardines usually combined with "soumbala," a local cooking powder

made of *Parkia biglobosa* seeds. Traps were set inside one factory (rice industry in Kirkissoye, KIR) and residential structures within 20 different localities. In each of them, a few rooms were investigated using 2-4 traps per room depending of available space. Full details regarding the localities investigated as well as the trapping effort for each type of traps are provided in Table 1.

**Table 1 Localities sampled during the 2020-2023 (P2) campaign.**

| Acronym | Locality | Type | Trapping effort | | | GPS | |
|---|---|---|---|---|---|---|---|
| | | | Wm | Sh | Total | Lat. (N) | Lon. (E) |
| AER | AEROPORT | RA | 112 | 104 | 216 | 13.51222 | 2.12138 |
| BOB | BOBIEL | RA | ? | ? | ? | 13.56076 | 2.09214 |
| **BOU** | **BOUKOKI** | **RA** | **595** | **162** | **757** | **13.53868** | **2.11395** |
| **DAR** | **DAR ES SALAM** | **RA** | **827** | **89** | **916** | **13.54693** | **2.09528** |
| **GAM** | **GAMKALLEY** | **RA** | **426** | **426** | **852** | **13.49388** | **2.12693** |
| **KAR** | **KARADJE** | **RA** | **78** | **36** | **114** | **13.49387** | **2.09441** |
| KIR | KIRKISSOYE | F | 28 | 17 | 45 | 13.495 | 2.10961 |
| KIR-1 | KIRKISSOYE 1 | RA | 6 | 3 | 9 | 13.48109 | 2.11656 |
| **KOT** | **KOIRA TEGUI** | **RA** | **30** | **40** | **70** | **13.58225** | **2.11442** |
| **CYA** | **CORNICHE YANTALA** | **RA** | **267** | **96** | **363** | **13.51262** | **2.09911** |
| LAC | LACOUROUSSOU | RA | 62 | 82 | 144 | 13.51222 | 2.12138 |
| LMO | LAMORDE | RA | ? | ? | ? | 13.50605 | 2.07266 |
| **GRM** | **LIBERTE** | **RA** | **97** | **62** | **159** | **13.51923** | **2.11518** |
| NYA | NIAMEY 2000 | RA | ? | ? | ? | 13.51427 | 2.1856 |
| PLT | PLATEAU | RA | ? | ? | ? | 13.54083 | 2.10273 |
| **TCH** | **TCHANGAREY** | **RA** | **30** | **102** | **132** | **13.58029** | **2.08317** |
| UAM | UAM | RA | ? | ? | ? | 13.49918 | 2.10535 |
| **WAD** | **WADATA** | **RA** | **35** | **32** | **67** | **13.51821** | **2.14419** |
| SNI | SONUCI | RA | 260 | 90 | 350 | 13.56429 | 2.07065 |
| LSG | LOSSA SOUNGOU | RA | ? | ? | ? | 13.54395 | 2.04551 |
| YAH | YANTALA-HAUT | RA | ? | ? | ? | 13.53435 | 2.08208 |

For each locality, the type of environment (RA= residential area, F= factory) as well as the trapping effort (i.e., the number of night-traps) using only Sherman (Sh), only locally wire mesh (Wm) and

both types of traps (Total) are indicated. Localities that could be statistically compared between the 2009-2013 (P1) and 2020-2023 (P2) campaigns are indicated in bold (see text for details).

## 2.3. Ten-year evolution of rodent communities

To facilitate a meaningful comparison of small mammal assemblages between P1 and P2 trapping periods, only sites that had been accurately monitored in terms of trapping effort and types of traps during both P1 and P2 campaigns were considered. This enabled a robust analysis of trap- and species-specific trapping success in nine given localities, namely BOU, CYA, DAR, GAM, GRM, KAR, KOT, TCH and WAD (Fig. 1)

**Fig 1:** Map showing sites available for a proper comparison between the two monitoring periods, namely P1 and P2. Localities are color-coded for each period.

## 2.4. Statistical analyses

Species-specific trapping successes retrieved during the P2 campaign were compared between Wm and Sh traps over all localities using t-tests. Since Wm and Sh traps were confirmed to display significantly different species-specific capture rates (see Results), both species and trap type were taken into account in all subsequent statistical comparisons.

Trap- and species-specific capture rates were compared over all available localities between P1 and P2 campaigns using pairwise Holm-adjusted Wilcoxon tests. When feasible (i.e., for localities where at least one individual was captured), trap- and species-specific capture rates were also compared at the locality level using the same procedure.

## 2.5. Ethical and data sharing

This study was part of a collaboration between Abdou Moumouni University of Niamey (UAM) and French Institute of Research for Sustainable Development (IRD) under the scientific partnership agreement number 301027/00. Explicit oral consent was obtained from each locality authorities as well as each household head where trapping was implemented. None of the small mammal species sampled here has protection status [29]. All captured animals were treated humanely, with due regard for their individual well-being, in accordance with the guidelines of the American Society of Mammalogists [30] (Sikes, Care, and Mammalogists 2016). Ethical clearance was obtained from the Ministry for Public Health of Niger Republic (permit N°054/2022/CNERS).

# 3. Results

## 3.1. Assessment of small mammal diversity and trap type effect

A total of 604 small mammals belonging to three rodent (*Rattus rattus, Mastomys natalensis* and *Mus musculus*) and one shrew (*Crocidura olivieri*) species were collected during the P2 campaign (Tab. 2). Black rats were the most abundant (N=315), followed by *M. natalensis* (N=213), house mice (N=46) and shrews (N=30).

**Table 2: Trapping effort and number of captures per locality for the 2020-2023 period (P2).**

| | Effort (trapping night) | | | catch number (%) | | | | |
|---|---|---|---|---|---|---|---|---|
| Locality | Wm | Sh | total | *Cro* | *Mna* | *Mmu* | *Rra* | Total |
| AER | 112 | 104 | 216 | 2 | 21 | 0 | 0 | 23 |

| | | | | | | | | |
|---|---|---|---|---|---|---|---|---|
| BOU | 595 | 162 | 757 | 6 | 17 | 0 | 118 | 141 |
| CYA | 30 | 40 | 70 | 1 | 14 | 0 | 21 | 36 |
| DAR | 267 | 96 | 363 | 15 | 1 | 0 | 80 | 96 |
| GAM | 827 | 89 | 916 | 0 | 80 | 0 | 2 | 82 |
| GRM | 97 | 62 | 159 | 0 | 0 | 35 | 26 | 61 |
| KAR | 426 | 426 | 852 | 0 | 3 | 0 | 20 | 23 |
| KIR | 78 | 36 | 114 | 0 | 0 | 0 | 8 | 8 |
| KIR-1 | 28 | 17 | 45 | 0 | 0 | 0 | 4 | 4 |
| KOT | 6 | 3 | 9 | 0 | 16 | 0 | 0 | 16 |
| LAC | 62 | 82 | 144 | 0 | 0 | 11 | 0 | 11 |
| SNI | 260 | 90 | 350 | 2 | 2 | 0 | 0 | 4 |
| TCH | 30 | 102 | 132 | 4 | 20 | 0 | 0 | 24 |
| WAD | 35 | 32 | 67 | 0 | 1 | 0 | 14 | 15 |
| BOB | ? | ? | ? | 0 | 0 | 0 | 5 | 5 |
| LAM | ? | ? | ? | 0 | 6 | 0 | 0 | 6 |
| LSG | ? | ? | ? | 0 | 25 | 0 | 0 | 25 |
| NYA | ? | ? | ? | 0 | 6 | 0 | 0 | 6 |
| PLT | ? | ? | ? | 0 | 0 | 0 | 5 | 5 |
| UAM | ? | ? | ? | 0 | 0 | 0 | 5 | 5 |
| YAH | ? | ? | ? | 0 | 1 | 0 | 7 | 8 |
| | **2853** | **1341** | **4194** | **30** | **213** | **46** | **315** | **604** |

"Wm" and "Sh" correspond to wire-mesh and Sherman traps, respectively. "Cro", "Mna", "Mmu" and "Rra" stand for *Crocidura olivieri*, *Mastomys natalensis*, *Mus musculus*, and *Rattus rattus*, respectively.

Comparison of capture rates during the P2 period unambiguously confirmed that Wm and Sh traps displayed significantly different results depending on the species considered. As such, Wm was found more effective at capturing *R. rattus* (Wm=263 captures / 4368 night-traps vs. Sh=29/2943; t=2.109, p=0.036), while Sherman traps were more suitable for capturing *M. musculus* (Wm=5/245 vs. Sh=91/221; t=-5.0341, p<0.001), *M. natalensis* (Wm=92/4368 vs. Sh=318/2945; t=-5.272, p<0.001) and *C. olivieri* (Wm=36/4368 vs. Sh=288/2943; t=-2.1753, p=0.031). These differences in species-specific capture rates depending of the trap type justified to conduct subsequent analyses by considering Wm and Sh traps separately.

## 3.2. Evolution of species-specific spatial distributions between P1 and P2 periods

Among the nine localities investigated during both the P1 and P2 campaigns (Tab. 1), *Rattus rattus* was captured in only two of them (CYA and GRM) during P1 (Fig. 2; Tab. 4), with a single and three captures, respectively, despite significant trapping efforts [16]. During the P2 period, the species was captured in seven of these same nine locations, namely BOU, CYA, DAR, KAR, GAM, GRM and WAD (Fig. 2; Tab. 4). When one considers captures obtained with both Sh and Wm traps, it appears that *M. natalensis* was found in all nine localities during both periods P1 and P2 (Fig. 2; Tab. 4). Despite the extensive trapping effort throughout the city ([16]; this study), *M. musculus* was captured only in GRM. However, Sh-based results unambiguously demonstrate that the species was abundant in this given locality during both P1 and P2 campaigns (Fig. 2; Tab. 4). Finally, the shrew *C. olivieri* was present in the P1 but asbent from the P2 datasets from several localities, namely, including three (KAR, GRM, and WAD).

In brief, from the spatial distribution perspective, *R. rattus* has been found in two then seven localities during the P1 and P2 campaigns, respectively, while *M. natalensis* was present in all nine localities during both periods. House mice were present but restricted to one single locality during the two campaigns. Shrews were found in six and four localities during P1 and P2, respectively.

**Fig 2:** Comparative maps of species relative abundances between the P1 (left panel) and P2 (right panel) campaigns as observed with either wire mesh (A) or Sherman (B) traps.

## 3.3. Comparison of species- and trap-specific capture rates between P1 and P2 campaigns

Taking trap type into consideration, we explored the differences in species-specific capture rates between P1 (2009-2013) and P2 (2020-2023) campaigns (see detailed results in Table 4). At the city scale, *Rattus rattus* capture rates exhibited a pronounced and significant increase for both wire mesh (0.2% during P1, to 11% during P2; p<0.001) and Sherman traps (0.4% to 2.4%; p=0.049). Upon examination of the locality level, the increase observed with Wm traps was significant for all (BOU, CYA, CAR, GRM and KAR; all p<0.037) but two localities (GAM and WAD, the latter showing only marginally significant increase, p=0.053). Systematic but non-significant increases were also observed for all localities with Sh-based data.

Conversely, a slight but highly significant decrease of *M. natalensis* capture rate was observed at the city scale for Sh traps (12.4% to 10.4%; p<0.001) between P1 and P2 periods. This decrease was lower and non-significant with Wm traps (2.3% to 2.2%; p=0.22). When looking at the locality level, no significant difference were observed with Wm between P1 and P2 for any localities (0.13<p<0.95) except one (KOT, p=0.01). However, highly significant differences were retrieved with Sh (all p<0.007), except for GAM (p=0.18), KOT (p=0.1) and TCH (p=0.79) which showed non-significant decreases.

**Table 4: Comparisons of trapping efforts, capture number and rates for each species between the P1 and P2 campaigns.**

| | | **wire-mesh traps** | | | | | | | **Sherman traps** | | | | | | | **Overall** |
|---|---|---|---|---|---|---|---|---|---|---|---|---|---|---|---|---|
| | | **2009-2013** | | | **2020-2023** | | | ***Stats*** | **2009-2013** | | | **2020-2023** | | | ***Stats*** | |
| ***Species*** | **Locality** | **NT** | **CN** | **CE** | **NT** | **CN** | **CE** | | **NT** | **CN** | **CE** | **NT** | **CN** | **CE** | | |
| *Rattus rattus* | BOU | 220 | 0 | 0 | 595 | 117 | 0.197 | p=0.00031*** | 229 | 0 | 0 | 162 | 1 | 0.006 | p=0.41$^{ns}$ | **292** |
| | CYA | 247 | 1 | 0.004 | 267 | 16 | 0.06 | p=0.0075** | 241 | 3 | 0.012 | 96 | 5 | 0.052 | p=0.53$^{ns}$ | |
| | DAR | 279 | 0 | 0 | 827 | 78 | 0.094 | p=0.0054** | 252 | 0 | 0 | 89 | 2 | 0.022 | p=0.53$^{ns}$ | |
| | GAM | 236 | 0 | 0 | 426 | 1 | 0.002 | p=0.39$^{ns}$ | 224 | 0 | 0 | 426 | 1 | 0.002 | p=0.39$^{ns}$ | |
| | GRM | 148 | 3 | 0.02 | 97 | 20 | 0.206 | p=0.036* | 157 | 4 | 0.025 | 62 | 6 | 0.097 | p=0.85$^{ns}$ | |
| | KAR | 337 | 0 | 0 | 78 | 20 | 0.256 | p<0.001*** | 318 | 0 | 0 | 36 | 0 | 0 | NA | |
| | WAD | 247 | 0 | 0 | 35 | 7 | 0.2 | p=0.053 | 250 | 0 | 0 | 32 | 7 | 0.219 | p=0.14$^{ns}$ | |
| | subtotal | 1714 | 4 | 0.002 | 2325 | 259 | 0.111 | p=1.3e-9*** | 1671 | 7 | 0.004 | 903 | 22 | 0.024 | p=0.049* | |
| *Mastomys natalensis* | BOU | 220 | 7 | 0.032 | 595 | 17 | 0.029 | p=0.13$^{ns}$ | 229 | 41 | 0.179 | 162 | 0 | 0 | p=0.0028** | **410** |
| | CYA | 247 | 9 | 0.036 | 267 | 14 | 0.052 | p=0.47$^{ns}$ | 241 | 52 | 0.216 | 96 | 0 | 0 | p=3e-4*** | |
| | DAR | 279 | 6 | 0.022 | 827 | 1 | 0.001 | p=0.14$^{ns}$ | 252 | 34 | 0.135 | 89 | 0 | 0 | p=0.00048*** | |
| | GAM | 236 | 3 | 0.013 | 426 | 7 | 0.016 | p=0.73$^{ns}$ | 224 | 20 | 0.089 | 426 | 73 | 0.171 | p=0.18$^{ns}$ | |
| | KAR | 337 | 10 | 0.03 | 78 | 3 | 0.038 | p=0.95$^{ns}$ | 318 | 39 | 0.123 | 36 | 0 | 0 | p=0.0013** | |
| | KOT | 135 | 2 | 0.015 | 30 | 7 | 0.233 | p=0.01* | 131 | 8 | 0.061 | 40 | 9 | 0.225 | p=0.1$^{ns}$ | |
| | TCH | 134 | 5 | 0.037 | 30 | 0 | 0 | p=0.17$^{ns}$ | 96 | 11 | 0.115 | 102 | 20 | 0.196 | p=0.79$^{ns}$ | |
| | WAD | 247 | 0 | 0 | 35 | 1 | 0.029 | p=0.35$^{ns}$ | 250 | 11 | 0.044 | 32 | 0 | 0 | p=0.0066** | |
| | subtotal | 1835 | 42 | 0.023 | 2288 | 50 | 0.022 | p=0.22$^{ns}$ | 1741 | 216 | 0.124 | 983 | 102 | 0.104 | p=9.7e-6*** | |
| *Crocidura olivieri* | BOU | 220 | 0 | 0 | 595 | 5 | 0.008 | p=0.045* | 229 | 2 | 0.009 | 162 | 1 | 0 | p=0.93$^{ns}$ | **54** |
| | CYA | 247 | 0 | 0 | 267 | 1 | 0.004 | p=0.34$^{ns}$ | 241 | 0 | 0 | 96 | 0 | 0 | NA | |
| | DAR | 279 | 1 | 0.004 | 827 | 15 | 0.018 | p=0.11$^{ns}$ | 252 | 0 | 0 | 89 | 0 | 0 | NA | |
| | GRM | 148 | 0 | 0 | 97 | 0 | 0 | NA | 157 | 3 | 0.019 | 62 | 0 | 0 | P=0.1$^{ns}$ | |
| | KAR | 337 | 2 | 0.006 | 78 | 0 | 0 | p=0.4$^{ns}$ | 318 | 12 | 0.038 | 36 | 0 | 0 | p=0.035* | |
| | KOT | 135 | 2 | 0.015 | 30 | 0 | 0 | p=0.42$^{ns}$ | 131 | 0 | 0 | 40 | 0 | 0 | NA | |
| | TCH | 134 | 0 | 0 | 30 | 0 | 0 | NA | 96 | 0 | 0 | 102 | 4 | 0.039 | P=0.3$^{ns}$ | |
| | WAD | 247 | 0 | 0 | 35 | 0 | 0 | NA | 250 | 6 | 0.024 | 32 | 0 | 0 | P=0.21$^{ns}$ | |
| | subtotal | 1747 | 5 | 0.003 | 1959 | 21 | 0.011 | p=0.085$^{ns}$ | 1674 | 23 | 0.014 | 619 | 5 | 0.008 | p=0.0046** | |
| *Mus musculus* | GRM | 148 | 0 | 0 | 97 | 5 | 0.0515 | p=0.14 | 157 | 61 | 0.3885 | 62 | 30 | 0.4839 | p~1 | **96** |
| | **Overall** | | **51** | | | **335** | | **0** | | **307** | | | **159** | | | **852** |

These data are organized according to trap types and localities. ”TN” and “N” stand for the number of trap-night (i.e., trapping effort) and the number of captures, respectively.

Although the capture rates of the indigenous species *C. olivieri* was always rather low, it was significantly greater during P1 than P2 when only Sh traps were considered at the city level (p=0.0046). However, no difference was observed between the two periods for Wm traps (p=0.085). At the locality level, these differences were less clear since there was a significant decrease for Sh only in KAR (from 3.8% to 0%; p=0.035) but no significant changes in the other localities (BOU, GRM, TCH and WAD, all p<0.1). A very slight increase was found significant with Wm traps in BOU (0% to 0.008%; p=0.045) while no change was observed in the other localities (CYA, DAR, KAR and KOT; all p>0.11).

House mice were captured during both P1 and P2 only in GRM, and no difference were observed in capture rates in this locality, whatever the type of traps used (both p>0.14).

# 4. Discussion

The originality of our study lies in the fact that the exact same trapping localities and sometimes sites were monitored at an interval of ten years in order to investigate possible changes in the compositional changes of commensal rodent communities within an African city, i.e. Niamey, Niger, in the context of the hypothesized gradual spread of two invasive species, namely the black rat *R. rattus* and the house mouse *M. musculus*.

As already highlighted in other studies (e.g., [16,27,28]), our data unambiguously confirm that different types of traps may display significantly different capture rates, thus once again showing the importance of trapping protocols for small mammal biodiversity assessment and analyses.

This appeared particularly critical here when addressing the inter-relationships between Wm-attracted rats vs. Sh-attracted *M. natalensis* and *M. musculus*.

We also show that the house mouse remains abundant in the only locality where it was already present ten years ago, but that it did not appear to expand massively throughout the city since then. During the 2020-2023 campaigns, this prolific species was also captured in LAC (Tab.2), a neighborhood located 1km apart from the first one (see Fig. 2), but in absence of LAC in the 2009-2013 dataset [16], we cannot argue about any spatial expansion. To our knowledge, there is no available data about the date of *M. musculus* introduction into Niamey. As a consequence, it is not known how long the species has been present in the city. Nevertheless, taking into account the invasion success of house mice in other major Sahelian cities (e.g., Dakar, Senegal: [21]; Bamako, Mali: Granjon et al., in press), we anticipate that this species will spread further within Niamey in the years and decades to come.

Conversely, our longitudinal survey unambiguously demonstrates that black rats have already expanded widely across the city, with its presence now ascertained in at least five new neighborhoods of our observational device (BOB, PLT, UAM, YAH et WAD). Our data thus clearly confirm Garba and colleagues' [16] hypothesis that *R. rattus* active bio-invasion is currently ongoing in Niamey. The species have spread for several kilometers across the urban landscape during the last ten years, thus showing a quite efficient colonization of the city. Interestingly, it has progressed mainly within the core city, while we did not observe it yet in the more peripheral and more recently urbanized areas, like KOT and TCH (Fig. 4). It is also noticeable that, although black rats were identified in Gamkalleye (GAM on Fig. 4) during the P2 campaign while they were not found there during the P1 one, this neighborhood may partly resist black rats' invasion.

Indeed, additional regular surveys conducted within this particular area in 2023 and 2024 tend to suggest that *R. rattus* does not increase neither in abundance nor in terms of infested households (our own unpublished data). Gamkalleye is one of the last traditionally built district of Niamey, with a large majority of houses still made of mud (i.e., the so-called "banco" material). Though densely built and populated, and located in the heart of Niamey, this now atypical urban area may keep rural-like features (e.g., sandy soils, mud-made walls, frequent presence of livestock breeding) that could at least partly favor the native *M. natalensis* over the recently introduced *R. rattus* species. This particular situation in Gamkalleye is still under longitudinal surveillance. However, the rapid dissemination of black rats in the core city on the one hand, and its absence or rareness in both peripheral (KOT, TCH) and traditionally built (GAM) zones on the other hand, strongly suggest that, in Niamey, black rat expansion primarily follows formal, usually hard-built, urbanization.

In addition to its expanding spatial distribution, our quantitative comparisons of trap-specific capture rates at the neighborhood levels suggest that *R. rattus* has also locally increased in relative abundance. In parallel, we found that *M. natalensis* tends to decrease in several settings where black rat was present. Together, these patterns strongly support the replacement of the former by latter species within Niamey, as already hypothesized by Garba and colleagues [16]. This native-to-invasive rodent displacement is further exemplified by the apparently achieved turnover observed in DAR where *M. natalensis* were abundant in 2009-2013 but are absent from the 2020-2023 dataset, while *R. rattus* was initially absent and are now dominant (Fig. 2).

Of course, such drastic changes in terrestrial small mammal faunas within cities have already existed, as shown by the extant rodent assemblages of large European or American cities where

only invasive, now cosmopolitan taxa (e.g., *Rattus rattus*, *R. norvegicus and M. musculus*) are present in hard-built environments. However, the process may be currently ongoing in Niamey, and this may raise several issues in a near future. In particular, proliferation of invasive animal reservoirs like rats may propagate new zoonotic pathogens or increase the prevalence of already circulating ones [31], especially within urban environments [11,32]. In several West African towns and villages, including in Niger, invasive black rats have already been suspected to fuel atypical trypanosomes dissemination [33,34]. Some authors have also pointed towards the risk of Seoul Orthohantavirus propagation in this part of the world following the spillover of this deadly virus from its usual reservoir *Rattus norvegicus* to its congeneric *R. rattus* [35]. Conversely, *M. natalensis* is known as the major reservoir for Lassa Mammarenavirus [36], although some rare instances of alternative hosts -which do not include *Rattus rattus*- have been described [37]. As a consequence, shifts in reservoir species assemblages as that described in the present study are expected to have important sanitary consequences in Sahelian cities: while some zoonotic pathogens may become rarer (e.g., Lassa virus), others may appear and cause human diseases that are essentially unknown, hence undiagnosed and untreated, in this region (e.g., Orthohantavirus fevers, atypical trypanosomiais). A wide panel of other infectious threats could also be impacted (e.g., leptospirosis, bartonellosis, helminth- and bacteria-mediated diarrhea, antibio-resistance evolution, etc.) and may deserve further attention.

Beyond health, rodents are also responsible for various socio-economic nuisances. In Niamey, a study on the knowledge and perception of rodents and rodent-associated issues revealed that 96.5% of 170 interviewed inhabitants from 18 different neighborhoods identified rodents as pests [38]. All over the city, people quoted rodent-induced damages on food and food stocks

(63.1%), houses infrastructures (47.3%) and furniture (19.5%) as well as clothes (16.8%), thus suggesting that rodents strongly reinforce economic vulnerability and represent a significant additional burden for inhabitants [38]. These data were collected from end of 2009 to May 2011, a period where *M. natalensis* was still over-dominant across most of Niamey [38]. However, black rats are also widely recognized as important pest rodents that may greatly damage crops, food stocks and household utensils [39]. As such, while rats progressively replace native *M. natalensis* within Niamey, we expect nuisances to persist but potentially to change in nature and/or intensity. Unfortunately, we are not aware of studies that have compared *M. natalensis* vs. *R. rattus* damages, or their perception by inhabitants, that would allow one to anticipate future socio-economic nuisances induced by the native-to-invasive rodent turnover documented here.

# 5. Acknowledgements

The long-term monitoring of rodents within the city of Niamey, Niger, has been supported since 2013 by the West African network of small mammal observatories (ObsMICE) coordinated by the French Institute for Sustainable Research (IRD; coord. A. Dalecky). This work was part of the SCARIA project (coord. G. Dobigny, Belmont Forum, “Pathways to Sustainability” call, 2021-2023) with the studies conducted in Niger funded by the South African National Research Fund. Abdoulaziz Danzabarma is supported by a PhD thesis bursary by the French Institute of Sustainable Development (IRD; convention signed between Mr Danzabarma, IRD and the Abdou Moumouni University of Niamey on the 22nd October 2022).

# References

1. Triunveri A, Scalise D. Rodents: Habitat, Pathology, and Environmental Impact. Nova Science Publishers, Incorporated; 2012.

2. Wilson DE, Reeder DM. Mammal species of the world: a taxonomic and geographic reference. JHU press; 2005.

3. Banks PB, Smith HM. The ecological impacts of commensal species: black rats, Rattus rattus, at the urban–bushland interface. Wildlife Research. 2015;42: 86–97. Available: https://www.publish.csiro.au/wr/WR15048

4. Negesse M, Yazezew D, Degefe G, Getachew G. Species composition, relative abundance and distribution of rodents in Wof-Washa Natural State Forest, Ethiopia. Global Ecology and Conservation. 2022;39: e02283. doi:10.1016/j.gecco.2022.e02283

5. Drake DR, Hunt TL. Invasive rodents on islands: integrating historical and contemporary ecology. Biol Invasions. 2009;11: 1483–1487. doi:10.1007/s10530-008-9392-1

6. Jones EP, Eager HM, Gabriel SI, Jóhannesdóttir F, Searle JB. Genetic tracking of mice and other bioproxies to infer human history. Trends in Genetics. 2013;29: 298–308. doi:10.1016/j.tig.2012.11.011

7. Cavia R, Cueto GR, Suárez OV. Changes in rodent communities according to the landscape structure in an urban ecosystem. Landscape and Urban Planning. 2009;90: 11–19. doi:10.1016/j.landurbplan.2008.10.017

8. Hima K, Houémenou G, Badou S, Garba M, Dossou H-J, Etougbétché J, et al. Native and invasive small mammals in urban habitats along the commercial axis connecting Benin and Niger, West Africa. Diversity. 2019;11: 238.

9. Diagne C, Ballesteros-Mejia L, Cuthbert RN, Bodey TW, Fantle-Lepczyk J, Angulo E, et al. Economic costs of invasive rodents worldwide: the tip of the iceberg. PeerJ. 2023;11: e14935. doi:10.7717/peerj.14935

10. Morand S, Bordes F, Blasdell K, Pilosof S, Cornu J-F, Chaisiri K, et al. Assessing the distribution of disease-bearing rodents in human-modified tropical landscapes. Journal of Applied Ecology. 2015;52: 784–794. doi:10.1111/1365-2664.12414

11. Dobigny G, Morand S. Zoonotic emergence at the animal-environment-human interface: the forgotten urban socio-ecosystems. Peer Community Journal. 2022;2. doi:10.24072/pcjournal.206

12. Bastos AD, Nair D, Taylor PJ, Brettschneider H, Kirsten F, Mostert E, et al. Genetic monitoring detects an overlooked cryptic species and reveals the diversity and distribution

of three invasive Rattus congeners in south Africa. BMC Genet. 2011;12: 1–18. doi:10.1186/1471-2156-12-26

13. Berthier K, Garba M, Leblois R, Navascués M, Tatard C, Gauthier P, et al. Black rat invasion of inland Sahel: insights from interviews and population genetics in south-western Niger. Biological Journal of the Linnean Society. 2016;119: 748–765.

14. Dalecky A, Bâ K, Piry S, Lippens C, Diagne CA, Kane M, et al. Range expansion of the invasive house mouse Mus musculus domesticus in Senegal, West Africa: a synthesis of trapping data over three decades, 1983–2014. Mammal Review. 2015;45: 176–190. doi:10.1111/mam.12043

15. Duplantier J-M, Granjon L, Bâ K. Répartition biogéographique des petits rongeurs au Sénégal. Journal of African Zoology. 1997;111: 17–26. Available: https://horizon.documentation.ird.fr/exl-doc/pleins_textes/pleins_textes_7/b_fdi_59-60/010026078.pdf

16. Garba M, Dalecky A, Kadaoure I, Kane M, Hima K, Veran S, et al. Spatial Segregation between Invasive and Native Commensal Rodents in an Urban Environment: A Case Study in Niamey, Niger. PLOS ONE. 2014;9: e110666. doi:10.1371/journal.pone.0110666

17. Granjon L, Artige E, Bâ K, Brouat C, Dalecky A, Diagne C, et al. Sharing space between native and invasive small mammals: Study of commensal communities in Senegal. Ecology and Evolution. 2023;13: e10539. doi:10.1002/ece3.10539

18. Kaleme PK, Bates JM, Belesi HK, Bowie RCK, Gambalemoke M, Kerbis-Peterhans J, et al. Origin and putative colonization routes for invasive rodent taxa in the Democratic Republic of Congo. African Zoology. 2011;46: 133–145. doi:10.1080/15627020.2011.11407486

19. Konečný A, Estoup A, Duplantier J-M, Bryja J, Bâ K, Galan M, et al. Invasion genetics of the introduced black rat (Rattus rattus) in Senegal, West Africa. Molecular Ecology. 2013;22: 286–300. doi:10.1111/mec.12112

20. Cazanove F. Le problème du rat dans le territoire de Dakar et dépendances. Annales de Médecine et de Pharmacie Coloniales. 1931. pp. 108–144.

21. Stragier C, Piry S, Loiseau A, Kane M, Sow A, Niang Y, et al. Interplay between historical and current features of the cityscape in shaping the genetic structure of the house mouse (*Mus musculus domesticus*) in Dakar (Senegal, West Africa). Peer Community Journal. 2022;2. doi:10.24072/pcjournal.85

22. Dossou H-J, Ahoyo Adjovi N, Houéménou G, Bagan T, Mensah G-A, Dobigny G. Invasive rodents and damages to food stocks: a study in the Autonomous Harbor of Cotonou, Benin. Biotechnol Agron Soc Environ. 2020 [cited 6 Jan 2024]. doi:10.25518/1780-4507.18326

23. Omudu EA, Ati TT. A survey of rats trapped in residential apartments and their ectoparasites in Makurdi, Nigeria. Res J Agric Biol Sci. 2010;6: 144–149. Available: http://www.aensiweb.net/AENSIWEB/rjabs/rjabs/2010/144-149.pdf

24. Taylor PJ, Arntzen L, Hayter M, Iles M, Frean J, Belmain S. Understanding and managing sanitary risks due to rodent zoonoses in an African city: beyond the Boston Model. Integrative Zoology. 2008;3: 38–50. doi:10.1111/j.1749-4877.2008.00072.x

25. Dobigny G. Inventaire et Biogéographie des rongeurs du Niger : nuisance aux cultures, implications dans certains problèmes de santé publique et vétérinaire. 2000. Available: https://hal.archives-ouvertes.fr/hal-00394489

26. Granjon L, Duplantier J-M. Les rongeurs de l'Afrique sahélo-soudanienne. IRD; 2009.

27. Badou S, Missihoun AA, Agbangla C, Gauthier P, Houéménou G, Dossou H, et al. Maritime international trade and bioinvasions: A three-year long survey of small mammals in Autonomous Port of Cotonou, Benin. Journal of Applied Ecology. 2023;61: 669–686. doi:10.1111/1365-2664.14557

28. Lucaccioni H, Granjon L, Dalecky A, Fossati O, Fur JL, Duplantier J-M, et al. From Human Geography to Biological Invasions: The Black Rat Distribution in the Changing Southeastern of Senegal. PLOS ONE. 2016;11: e0163547. doi:10.1371/journal.pone.0163547

29. IUCN. The IUCN Red List of Threatened Species. In: IUCN Red List of Threatened Species [Internet]. [cited 27 Nov 2024]. Available: https://www.iucnredlist.org/en

30. Sikes RS, Care A, Mammalogists UC of the AS of. 2016 Guidelines of the American Society of Mammalogists for the use of wild mammals in research and education. Journal of mammalogy. 2016;97: 663–688. Available: https://academic.oup.com/jmammal/article-abstract/97/3/663/2459909

31. Young HS, Parker IM, Gilbert GS, Guerra AS, Nunn CL. Introduced species, disease ecology, and biodiversity–disease relationships. Trends in Ecology & Evolution. 2017;32: 41–54. Available: https://www.cell.com/trends/ecology-evolution/fulltext/S0169-5347(16)30173-2

32. Blasdell KR, Morand S, Laurance SGW, Doggett SL, Hahs A, Trinh K, et al. Rats and the city: Implications of urbanization on zoonotic disease risk in Southeast Asia. Proc Natl Acad Sci USA. 2022;119: e2112341119. doi:10.1073/pnas.2112341119

33. Tatard C, Garba M, Gauthier P, Hima K, Artige E, Dossou D, et al. Rodent-borne Trypanosoma from cities and villages of Niger and Nigeria: A special role for the invasive genus Rattus? Acta Tropica. 2017;171: 151–158. Available: https://www.sciencedirect.com/science/article/pii/S0001706X16311019

34. Dobigny G, Gauthier P, Houéménou G, Dossou HJ, Badou S, Etougbétché J, et al. Spatio-temporal survey of small mammal-borne Trypanosoma lewisi in Cotonou, Benin, and the potential risk of human infection. Infection, Genetics and Evolution. 2019;75: 103967. Available: https://www.sciencedirect.com/science/article/pii/S1567134819301856

35. Castel G, Filippone C, Tatard C, Vigan J, Dobigny G. Role of seaports and imported rats in Seoul hantavirus circulation, Africa. Emerging Infectious Diseases. 2023;29: 20. Available: https://www.ncbi.nlm.nih.gov/pmc/articles/PMC9796207/

36. Lecompte E, Fichet-Calvet E, Daffis S, Koulémou K, Sylla O, Kourouma F, et al. Mastomys natalensis and lassa fever, West Africa. Emerging infectious diseases. 2006;12: 1971. Available: https://www.ncbi.nlm.nih.gov/pmc/articles/PMC3291371/

37. Olayemi A, Cadar D, Magassouba N, Obadare A, Kourouma F, Oyeyiola A, et al. New hosts of the Lassa virus. Scientific reports. 2016;6: 25280. Available: https://www.nature.com/articles/srep25280

38. Garba M, Kane M, Gagare S, Kadaoure I, Sidikou R, Rossi J-P, et al. Local perception of rodent-associated problems in Sahelian urban areas: a survey in Niamey, Niger. Urban Ecosyst. 2014;17: 573–584. doi:10.1007/s11252-013-0336-x

39. Soarimalala V, Randriamanana JP, Onjaniaina G. Les rats dans le monde rural du Centre-est et du Centre-sud de Madagascar: Dommages causés et systèmes de contrôle. Malagasy Nature. 2019;13: 125–51. Available: http://www.vahatra.mg/volume13/MN13_10_Soarimalala%20et%20al.pdf

Fig 2 Click here to access/download;Figure;Fig 2.tif

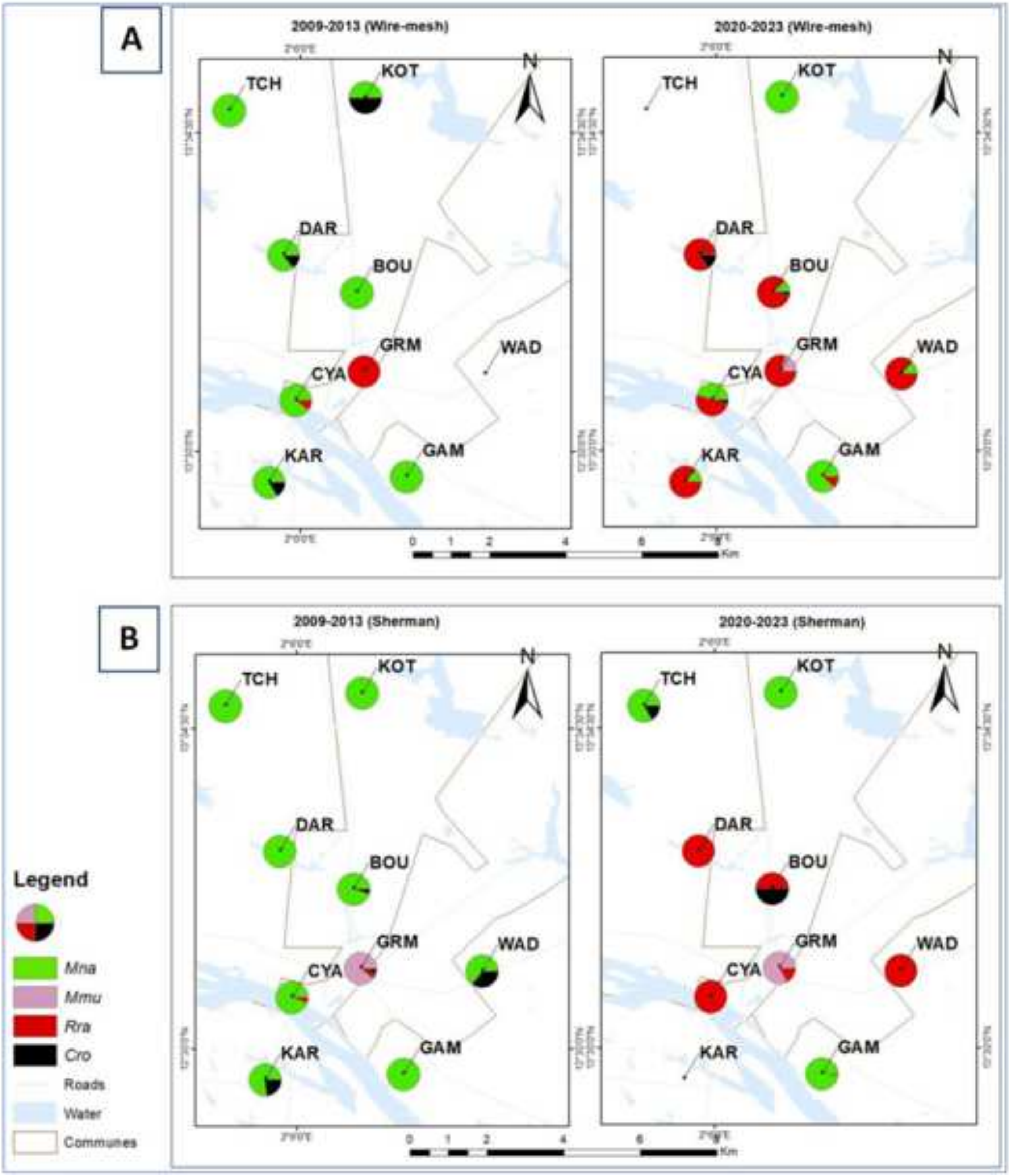

Fig 1

Click here to access/download;Figure;Fig 1.tif

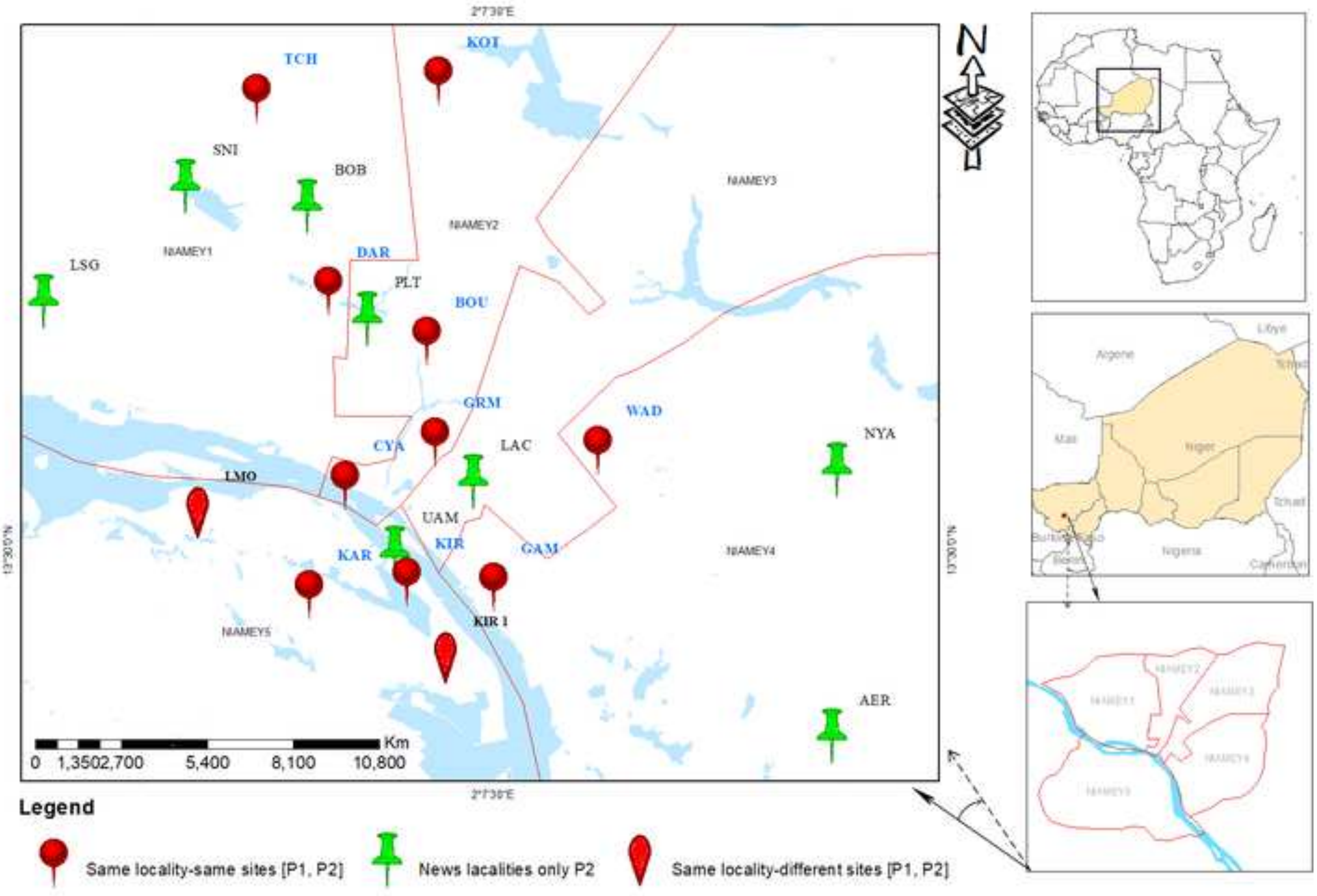

Click here to access/download
**Supporting Information**
Ethic_English.pdf
