# Supplementary material for: Native-to-invasive rodent species turn-over within African cities: The example of Niamey, Niger
Source: PLoS One. 2025 Jul 22;20(7):e0325427. doi: 10.1371/journal.pone.0325427 (PMC12282928; doi:10.1371/journal.pone.0325427)
Supplement: Attachment — Submitted filename: Response to Reviewers.docx [file pone.0325427.s005.docx]

Université Abdou Moumouni de Niamey

Faculté des Sciences et Techniques

Département de Biologie

BP. 10662, Niamey, NIGER

Tel: 227 + 98136998

Email: [az.danzabarma@gmail.com](mailto:az.danzabarma@gmail.com)

*Niamey, Saturday 12^th^ avril 2025*

Dear *Plos One* Editorial Board,

Please find attached our revised manuscript on “Native-to-invasive rodent species turn over within African cities: an example in Niamey, Niger” by Abdoulaziz IBRAHIM DANZABARMA and colleagues.

We would like to thank all the editorial board of the journal and the reviewers of the manuscript for the very positive comments. Also, we would like to apologize for the delay in replying, this is due to the too busy schedule for the main authors.

We hope that we have replied to all of the reviewer’s comments. So, we have revised both the text, Figures and Tables in accordance of their comments and suggestions.

Please find below this letter, as replies to both editorial board and the reviewers, our detailed responses point-by-point.

Kind regards with thanks and all the best wishes

On behalf of the manuscript’s authors

Aziz IBRAHIM DANZABARMA

**Manuscript #** **PONE-D-24-55809: Native-to-invasive rodent species turn-over within African cities: the example of Niamey, Niger**

COMMENTS FROM ACADEMIC EDITOR: Dr Javier delBarco-Trillo

Academic Editor

Comments to the Author:

Dear Dr. Ibrahim Danzabarma,

Thank you for submitting your manuscript to PLOS ONE. After careful consideration, we feel that it has merit but does not fully meet PLOS ONE’s publication criteria as it currently stands. Therefore, we invite you to submit a revised version of the manuscript that addresses the points raised during the review process. Please submit your revised manuscript by Apr 06 2025 11:59PM. If you will need more time than this to complete your revisions, please reply to this message or contact the journal office at [plosone@plos.org](mailto:plosone@plos.org). When you're ready to submit your revision, log on to <https://www.editorialmanager.com/pone/> and select the 'Submissions Needing Revision' folder to locate your manuscript file.

Please include the following items when submitting your revised manuscript:

- A rebuttal letter that responds to each point raised by the academic editor and reviewer(s). You should upload this letter as a separate file labeled 'Response to Reviewers'.
- A marked-up copy of your manuscript that highlights changes made to the original version. You should upload this as a separate file labeled 'Revised Manuscript with Track Changes'.
- An unmarked version of your revised paper without tracked changes. You should upload this as a separate file labeled 'Manuscript'.

If you would like to make changes to your financial disclosure, please include your updated statement in your cover letter. Guidelines for resubmitting your figure files are available below the reviewer comments at the end of this letter.

If applicable, we recommend that you deposit your laboratory protocols in [protocols.io](http://protocols.io/) to enhance the reproducibility of your results. Protocols.io assigns your protocol its own identifier (DOI) so that it can be cited independently in the future. For instructions see: [https://journals.plos.org/plosone/s/submission-guidelines#loc-laboratory-protocols](https://track.editorialmanager.com/CL0/https:%2F%2Fjournals.plos.org%2Fplosone%2Fs%2Fsubmission-guidelines%23loc-laboratory-protocols/1/010f019524a12fb9-d32ac847-82a0-4764-a9b3-87d26493eee9-000000/azr3o0-AFwrvD5_j65-IhkcPnTj-0SuCe4RTbRvS3zM=199). Additionally, PLOS ONE offers an option for publishing peer-reviewed Lab Protocol articles, which describe protocols hosted on [protocols.io](http://protocols.io/). Read more information on sharing protocolsat [*https://plos.org/protocols?utm_medium=editorialemail&utm_source=authorletters&utm_campaign=protocols*](https://plos.org/protocols?utm_medium=editorialemail&utm_source=authorletters&utm_campaign=protocols)*.*

*Response #1: We are very grateful to the Editor and the three reviewers for their thorough and constructive comments, which have greatly contributed to improving the scientific quality and clarity of our manuscript. We are pleased to know that our work can still be considered for publication in PLOS ONE, provided that we address the concerns and suggestions raised. In the revised version of the manuscript (file labeled 'Manuscript'), we have carefully taken into account all comments from the reviewers and made the necessary revisions.* *All changes are highlighted using track changes. Below, we provide point-by-point detailed response to each comment. Please note that all references to line numbers correspond to those in the revised manuscript mentioned above (after the inclusion of track changes modifications). We hope that these revisions will meet the expectations of the Editor and reviewers, and that our manuscript will now be suitable for publication in PLOS ONE.*

Journal requirements:

When submitting your revision, we need you to address these additional requirements.

1. Please ensure that your manuscript meets PLOS ONE's style requirements, including those for file naming. The PLOS ONE style templates can be found at

<https://journals.plos.org/plosone/s/file?id=wjVg/PLOSOne_formatting_sample_main_body.pdf> and

[https://journals.plos.org/plosone/s/file?id=ba62/PLOSOne_formatting_sample_title_authors_affiliations.pdf](https://track.editorialmanager.com/CL0/https:%2F%2Fjournals.plos.org%2Fplosone%2Fs%2Ffile%3Fid=ba62%2FPLOSOne_formatting_sample_title_authors_affiliations.pdf/1/010f019524a12fb9-d32ac847-82a0-4764-a9b3-87d26493eee9-000000/lGYnLjEoAf8b5q_RylbAjtUNYVoe2u2i0Hx1mfOvTBw=199).

*Response #2: We have revised our manuscript to fully comply with PLOS ONE’s formatting guidelines, following the templates provided in the links shared. The file names have been updated to meet the journal’s style requirements.*

2. Please provide captions for Fig. 4 in your manuscript.

*Response #3: We thank the editorial team for noting this. After careful verification, we confirm that our manuscript contains only two figures (Figures 1 and 2). The mention of "Figure 4" was an oversight, and we have now removed this mention from the revised version of the manuscript.*

3. In the ethics statement in the Methods, you have specified that verbal consent was obtained. Please provide additional details regarding how this consent was documented and witnessed, and state whether this was approved by the IRB.

*Response #4: The section “Ethics Statement” has been revised so and more details are provided regarding how verbal consent was obtained. The updated statement can be found in the Section “Ethical and data sharing” (lines 173–181) and now reads as follows: “**Prior to rodent trapping per se, the study’s objectives, methodology, and potential implications were thoroughly explained to neighborhood chiefs, who appointed local representatives to facilitate communication with household heads. Verbal informed consent was explicitly obtained from each household head before any trapping activities took place, ensuring full compliance with ethical research standards and community collaboration”. A further point of relevance regarding Niger is as follows: In Niger, the Ministry of Public Health (MSP) is the only authorized body to grant permits for conducting studies involving animals, in accordance to national legislation (Decree No 2016-000092/PRN/MSP). Therefore, in this country, the MSP is legally equivalent to an Animal Care and Use Committee or an Animal Ethics Committee. Accordingly, the necessary authorization for our study was obtained directly from the MSP, following the required legal procedure in Niger, as now explicitly stated in the Section “Ethical and data sharing”.*

4. We note that the grant information you provided in the ‘Funding Information’ and ‘Financial Disclosure’ sections do not match. When you resubmit, please ensure that you provide the correct grant numbers for the awards you received for your study in the ‘Funding Information’ section.

*Response #5: We have reviewed and corrected the grant information to ensure consistency between the ‘Funding Information’ and the ‘Financial Disclosure’ sections. The correct grant numbers and sources of funding have been taken into account and added to the corresponding section during the resubmission.*

5. Thank you for stating the following financial disclosure: [GD, SCARIA Project funded by Belmont Forum AID, Phd Thesis scholarship funded by IRD]. Please state what role the funders took in the study.  If the funders had no role, please state: ""The funders had no role in study design, data collection and analysis, decision to publish, or preparation of the manuscript."" If this statement is not correct you must amend it as needed. Please include this amended Role of Funder statement in your cover letter; we will change the online submission form on your behalf.

*Response #6: As requested, we have added the following statement to clarify the role of funders: “The funders had no role in study design, data collection and analysis, decision to publish, or preparation of the manuscript.” This statement now appears in the ‘Financial Disclosure’ section, too.*

6. We note that your Data Availability Statement is currently as follows: [All relevant data are within the manuscript and its Supporting Information files.] Please confirm at this time whether or not your submission contains all raw data required to replicate the results of your study. Authors must share the “minimal data set” for their submission. PLOS defines the minimal data set to consist of the data required to replicate all study findings reported in the article, as well as related metadata and methods (<https://journals.plos.org/plosone/s/data-availability#loc-minimal-data-set-definition>).

For example, authors should submit the following data:

- The values behind the means, standard deviations and other measures reported;

- The values used to build graphs;

- The points extracted from images for analysis.

Authors do not need to submit their entire data set if only a portion of the data was used in the reported study.

If your submission does not contain these data, please either upload them as Supporting Information files or deposit them to a stable, public repository and provide us with the relevant URLs, DOIs, or accession numbers. For a list of recommended repositories, please see

<https://journals.plos.org/plosone/s/recommended-repositories>.

If there are ethical or legal restrictions on sharing a de-identified data set, please explain them in detail (e.g., data contain potentially sensitive information, data are owned by a third-party organization, etc.) and who has imposed them (e.g., an ethics committee). Please also provide contact information for a data access committee, ethics committee, or other institutional body to which data requests may be sent. If data are owned by a third party, please indicate how others may request data access.

*Response #7: We confirm that all relevant raw data required to replicate the results of this study are either presented in the main text or included in the tables, making them fully available within the manuscript.*

**Reviewer # 1**

REVIEWER REPORT FOR THE AUTHORS

Reviewer #1: Dear authors, congratulations on your work! It was a pleasure to read it, very well explained and justified. I just miss a bit of attention in the graphs, I think they could be improved. Also, I suggested in the comments transforming a table into a barplot so we can see more clearly the differences in your results, since you just tested differences with t-tests. I think these would largely improve your work.

*Response #8*: *We would like to thank the reviewer #1 for his.her positive and encouraging feedback on our work and manuscript. We are very grateful that his.her found our work well explained and justified. We appreciate his.her constructive suggestions, which we hope have helped us to improve the clarity and quality of our article. All of his.her comments have been considered and taken into account in the revised version of the manuscript. Thank you for finding below our point-by-point responses to his.her comments.*

- about the comment: "I just miss a bit of attention in the graphs, I think they could be improved."

Response #8.1: *We fully agree that our graphs quality can be improved for more readability and visual comfort. Accordingly, we have increased the font size of axes titles and labels, enhanced the color contrasts, and adjusted the resolution to ensure increased clarity. These changes have been applied to the two figures of the manuscript (see Figures 1 and 2).*

- For the suggestion to transform a table into a barplot for better visualization of differences: "I suggested in the comments transforming a table into a barplot so we can see more clearly the differences in your results, since you just tested differences with t-tests. I think these would largely improve your work."

*Response #8.2: We agree with the interest of transforming the table into a barplot to better visualize differences. However, we think that it would be more useful to keep the table because it allows us to provide pieces of information that cannot be easily and clearly shown in a barplot, while these pieces of information are requested by PLoS ONE editorial policy to permit reproduction of analyses by authors (see above): these include number of trap-nights and captures as well as and capture rates, which form the basis of our quantitative analyses and results (see below). However, for more clarity and as suggested by Reviewer 3, the table legend has been improved by giving more details on variables.*

*Finally, at line 26-, the reviewer#1 asked: "Why is this -R.rattus- abbreviated and Mastomys natalensis is not?" This was an omission. According to standard conventions, species names should not be abbreviated upon their first mention in the text. This applies to Rattus rattus as well as the other species. We have now corrected this for consistency (see line 26), ensuring that the full species names are used at their first appearance.*

**Reviewer # 3**

REVIEWER REPORT FOR THE AUTHORS

Reviewer #3: In ‘Native-to-invasive rodent species turn-over within African cities: the example of Niamey, Niger’, the authors compare small mammal presence and abundance at 9 localities in Niamey sampled about 10 years apart and conclude that R. rattus presence is expanding in the city, while M. natalensis is less abundant but equally prevalent. This is a simple study with sparsely described methods that makes a small contribution to our understanding of the expansion of an invasive rodent in urban areas over time; however, the statistical analyses are such that strong conclusions cannot be made. In addition, there seems to be a missed opportunity here to delve more deeply into the urban conditions specifically associated with Rattus expansion/abundance or to tease apart potential drivers of this expansion. Some detailed comments follow?

*Response #9*: *We would like to thank reviewer #3 for taking the time to proofread our article. His.her contribution through the remarks and comments made, has been used to improve the quality of the revised manuscript. Before giving our point-by-point responses to his.her comments, please let us pick up on the global feedback.*

*First, we concede that the design of our study is simple. However, we also believe that it is scientifically sound and robust, that it is adapted to our questions and proved to be valuable to answer them unambiguously. Note that gathering comparable datasets ten years apart is, by itself, scientifically challenging: actually, we are not aware of such long-term longitudinal comparisons of urban rodent communities that allow one to address species-specific turn-over at both a city- and household-scales.*

*Second, we do not agree that our methodology was scarcely described: once again, though simple, all field and statistical procedures were provided so that analyses can be evaluated and re-implemented. As they stand, the provided comments by reviewer #3 do not point towards precise caveats that we could possibly address. Nevertheless, in case we would have indeed overlooked something, we kindly invite reviewer #3 to list the particular aspects of the materials and methods that, according to him.her, would deserve to be further detailed.*

*Third, echoing our first point, we believe that our contribution is based on simple (but robust) data and methods, but that it is not necessarily “small” since long-term longitudinal studies directly observing ongoing expansion of an invasive rodent species within a city, as well as the accompanying native-to-invasive replacement patterns are very rare. This is probably due to the challenge of conceiving, setting up and maintaining durable monitoring devices over so long periods (here, ten years!).*

*Last, thanks for suggesting us to deepen the urban conditions specifically associated with Rattus expansion/abundance, and/or to tease apart potential drivers of this expansion. Accordingly, the discussion section was enriched in order to address a bit further these particular aspects.*

Line 38 – “rats and mice” are not species names and can encompass a wide range of animals with different life history traits. It would be preferrable to be specific here.

*Response #10*: *The phrase "rats and mice" has been modified accordingly (see line 39).*

Line 42 – The provided references do not support the claim that the continent of Africa is any different from South America (for example) in terms of colonization of invasive rodents. This study doesn’t require that Africa be unique in this way for the paper to be of interest, so can be removed.

*Response #11*: *We are not entirely sure we fully understand the observation, but here is our response. The references [12-19] bring support to the statement that the colonization of Africa by invasive rodents is still ongoing and not yet complete – with no comparison with other continents. We here aimed at highlighting that Sahelian cities like Niamey were relevant case studies of ongoing invasion, thus reinforcing the significance of the paper. For this reason, we have decided to retain this statement and these important references.*

Line 49 – Please clarify what “essentially house mice” means.

*Response #12*: *The phrase "essentially house mice" has been replaced with "mostly house mice", thus making our specific reference to Mus musculus clearer. (see line 50)*

Line 50 – Please use consistent numbers of significant digits for % throughout

*Response #13*: *We have decided to report percentages and ratios with no more than one decimal, as we believe higher precision is not really meaningful given the nature of our data, namely ratios to express trapping success, (species-to-community) relative abundance, etc (see lines 51-52). For probabilities, however, we have maintained two or three decimal places depending on the level of precision required by the analytic results and/or statement (e.g., 0.049 is kept to three decimal places because it is close to our 0.05 statistical significance threshold, while 0.22 remains at two decimal places since further precision is useless to document better statistical non significance), as shown in lines 225–233 for the examples of p-values.*

Line 55 – Suggest referring to the map in Figure 1 here.

*Response #14: According to the journal’s guidelines, it is not possible to cite the Figure 1 in the introduction section because each cited figure must appear immediately after the first paragraph where it is mentioned. Yet, Figure 1 is specifically relevant for the "Study Area" section, but not for the introduction.*

Lines 56-67 – Please indicate which (if any) of these studies were performed by authors of the current MS – especially Ref 16, which does seem to include some of the same names. It’s a bit confusing the way this part and the beginning part of the methods are written as to if this study followed the same methodology consistently between studies or just attempted to replicate the methodology as it appeared in the original publications.

*Response #15: We confirm that Reference 16 was conducted by some of the authors of the current manuscript (K. Hima, M. Garba and G. Dobigny), thus having greatly facilitated the strict reapplication of the same experimental procedures ten years later. As stated in the text, using the same protocols has ensured consistency and comparability of the data collected over time.*

Methods – Please provide details about what happened to the rodents during and after trapping. This is a significant oversight in methodology and should include (at a minimum) how long the traps were open for, how often they were checked, how they were handled/sampled, if they were sedated, if they were euthanized, and if so, under which procedure?

*Response #16: Detailed information has been now added to the revised version of the manuscript (lines 120-128). It now reads as follows: “Rodents were captured and brought alive to the laboratory in their own traps immediately after capture. They were sacrificed within the next 3-4 hours following cervical dislocation in accordance with the guidelines of the American Society of Mammalogists about rodent handling, process and euthanasia (Mills et al., 1995; Sikes et al., 2011). Observations and measurements (weight, body measurements, sex, reproductive activity, possible ecto-parasites) were systematically carried out afterwards. Tissue samples were also collected for future molecular analyses (species-specific barecoding identification, pathogens detection, population genetics, etc)*”.

Lines 81-82 – This sentence is hard to follow as written. Please rephrase. Please also include here which/how many of the original localities you retained in the current study.

*Response #17: We agree that the sentence “In their study, 26 localities, including 18 neighborhoods, were sampled with an effort of 4 to 18 sites per locality, 7 market gardens as well as 2 industrial areas.” was unclear. As a consequence, it was reworded and now reads: “In their study, 26 localities were sampled, including 18 residential neighborhoods, 6 market gardens and 2 industrial areas, with an effort of sampling 2 to 4 sites per locality (see lines 83-85). For the current study, we retained 9 of their residential neighborhoods, namely (BOU, CYA, DAR, GAM, GRM, KOT, TCH, KAR and WAD)”.*

*Regarding sites investigated in both studies, they are also presented in Table 1 (In bold) for further clarity, and all the details are shown on Map of Figure 1 as well, with additional information provided in the figure legend (see legend lines 144-148: “****Fig 1:*** *Study area map of Niamey, Niger, showing sampled localities across the two studies (2010-2013 and 2020-2023). The red lines denote the legal boundaries of the five municipal districts. Watercourses are shown in blue. Red dots represent localities sampled at the exact same sites during both studies; yellow dots indicate localities that were sampled during both studies but at different sites; and green dots show localities that were sampled only during the second study (Period 2, i.e., 2020-2023).*

Line 94: “Each session consisted in three” should read “Each session consisted of three”

*Response #18:* *We thank the reviewer for this suggestion. However, the entire sentence has been rewritten to provide greater clarity regarding the trapping process. The fully revised sentence now reads: "In each investigated spaces (courtyards, grain mills, dwellings and/or business premises such as stores or workshops), two to four traps (depending on the space available within one given space) were set during three consecutive nights. They were set in the afternoon, and checked in the next morning: successful traps were replaced by a new baited traps laid at the exact same place, while unsuccessful traps were rebaited and reset for another night.". (see line 98-104).*

Line 94-95: Was the sampling period the same for P1?

*Response #19: This remark does not require any change to the text. Nevertheless, in order to answer reviewer #3’s question, it should be noted that the exact timing of the trapping sessions during the P1 (2010-2013) and P2 (2020-2023) campaigns did not fully overlap in terms of months or seasons. For P1 and P2, the sampling spanned two and three years, respectively. This means that for a given period (either P1 or P2), not all localities were sampled within the same year and within the same month/season – which, considering the large dataset used in our study (i.e., 987 individuals among 52 different intra-Niamey neighborhoods during the 2010-2013 campaign, see Garba et al., 2014, and 604 captures among 21 different intra-Niamey neighborhoods during the 2020-2023 campaign) would not have been feasible. However, our study aims at investigating wide-scale changes on the long-term (i.e. change in rodent communities within a ten year long laps of time); moreover, urban rodents are not known to undergo annual migrations that would result in drastic changes in their seasonal distribution. As a consequence, pooling trapping results over seasons of the same 2-3 successive years is not expected to influence fauna census when comparing the 2010-2013 and 2020-2023 periods (i.e., P1 and P2, respectively).*

Table 1 – Why do some localities show? for trapping effort?

*Response #20: The "?" symbols in Table 1 correspond to localities where trapping effort was not properly noted. They usually correspond to additional sampling opportunities outside our usual standard protocols, e.g. traps put at colleagues or friends’ places where we cannot ensure that they were correctly set and/or monitored all nights. Of course, these data are only informative in terms of presence of species that were indeed collected, but they are not valuable to explore absence or relative abundance; hence they cannot be, and were not used for statistic comparisons in our study. For clarity, "?" were replaced by "NA"(Not Applicable) in Table 1 (see Table 1).*

Lines 113-117 – Given that the authors have decided to only use localities that were sampled in both P1 and P2 in the analysis, why were new localities sampled in P2? Is it necessary to include those new localities in this MS? If so, that should be clearly justified.

*Response #21: One of the main objectives of the study was indeed to compare the evolution of rodent communities ten years apart, namely between the 2010-2013 (P1) and 2020-2023 (P2) periods. Consequently, as acknowledged by reviewer #3, only localities that were rigorously investigated during the two periods were included in the analyses addressing this particular issue. However, additional data from extra localities were collected across Niamey during the P2 period, thus allowing us to document further the extant distributions of native and invasive species observed to date in Niamey. We believe it is important to make them available since they could be of great interest for future research groups or programs that would build on our own data, and that would pursue the study of invasion processes in Niamey. In addition, such census data are not so frequent within African cities, and may be of interest for other purposes, including eco-evolutionary meta-analyses, niche modeling, zoonotic pathogen screenings, etc. For these reasons, we do not see any valuable reason not to provide them in the present paper.*

Figure 1 – The maps in the small boxes to the right are very hard to see and interpret, and in general the figure quality is quite poor.

*Response #22: We concede that the quality and readability had to be improved for the maps in Figure 1, as well as for all other figures, to enhance clarity and to ensure better visibility. This is why Figure 1 has been enlarged and is now presented with a higher graphical resolution* *(see Figure 1).*

Lines 120-128 – There is not enough detail about statistical methods here. In particular, how was trapping effort accounted for?

*Response #23: Statistic comparisons all relied on capture success rates, more precisely on species- (and/or trap-) specific capture rates, which corresponds to the number of individuals captured (per trap type) divided by the number of trap-nights (= the corresponding trapping effort). This allowed us to account for variations in trapping effort across localities and/or periods in all statistical investigations; in no instance were statistical tests performed in raw numbers of capture which, of course, strongly depend on trapping effort. In order to ensure clarity, a statement was added in the section “Statistical Analyses” (see lines 152-154). (As stated above (see Response #20), newly added localities for which trapping effort was not available were not included in statistical analyses.*

*Statistical analyses were carried out on these capture rates (thus being systematically loaded by trapping effort) only, with appropriate tests (paired t-tests and Wilcoxon signed-rank tests) applied for comparisons between trap types and periods, as explained in the text.*

Lines 130-138 – I do not see evidence that the Ministry for Public Health of Niger Republic is the equivalent of an Animal Care and Use Committee or Animal Ethics committee. Please include details regarding the capture, handling and release/euthanasia of animals in the methods and/or include the approved protocol as supplementary material. Please note that PLoS Instructions for publications involving animal research REQUIRE details about the IACUC committee used, as well as details regarding anesthesia and euthanasia, to be included.

*Response #24: In Niger, the Ministry of Public Health (MSP) is the only authorized body to grant permits for conducting studies involving animals, in accordance to national legislation (Decree No 2016-000092/PRN/MSP). Therefore, the MSP is legally equivalent to an Animal Care and Use Committee or an Animal Ethics Committee. Accordingly, the necessary authorization for our study was obtained directly from the MSP, following the required legal procedure in Niger, as stated in the Section “Ethical and data sharing”. Details regarding the 'Capture, Handling, Release, and Euthanasia of Animals' have now been added to the methodology section (see lines 120-128).*

Line 160-175 – Given that this section refers to Figure 2 repeatedly, I’m confused as to why Figure 2 shows abundance data while this section only refers to presence/absence data. If abundance data is relevant, please discuss in the text or adjust the figure to show the data as it’s presented in the text.

*Response #24:* *We thank reviewer #3 for this observation and appreciate the opportunity to clarify this point. We recognize the concern about consistency between the text and Figure 2. However, we believe that displaying abundance data provides additional valuable information beyond simple presence/absence data, even if not directly used in statistical analyses. Since presence/absence can easily be inferred from abundance data (where an abundance of 0 indicates absence), we have chosen to retain the original figure while ensuring that the text now better explains how the data should be interpreted.*

Line 162 – Please specify what “significant trapping efforts” means.

*Response #25:* *Thank for your request for clarification. By “significant trapping efforts,” we refer to a high number of trap-nights, considering the number of captures obtained. This is not a statistical term, but rather a description of the substantial amount of trapping conducted but that, despite this, did not allow to retrieve the species considered in this section.*

Line 165 – Why does it only “appear” that M. natalensis was found in 9 localities? Either they were found or they were not.

*Response #26: We appreciate the reviewer’s comment, and we have revised the sentence for clarity. The updated version now reads: "When one considers captures obtained with both Sh and Wm traps, M. natalensis was found in eight localities during both periods P1 and P2." (see lines 204-206).*

Line 176 – There is not enough information in the Figure 2 caption to be able to interpret the figure. Details about what the pie charts mean and how they were calculated is missing. It is also unclear how trap efforts varied between the P1 and P1 sessions, and if that was accounted for in abundance estimates? Are these per site or per locality?

*Response #27:* *Following the modification of Figure 2 to show only presence/absence data, trapping effort is no longer taken into account in this figure. However, detailed information on trapping effort, number of captures, and trap success is fully provided in Table 3.*

Figure 2 - Rather than display the abundances of the two trap types differently, I would suggest combining them and showing site-specific relative community abundances in one pie chart per site to highlight the changes between P1 and P2 more clearly. Although the analyses were performed separately due to differences in catch rate, presenting the small mammal composition at a site is not best displayed by separating them in the figure.

*Response #27: Thank you for your suggestion. We have decided to retain the abundance data for the figure, for the reasons already outlined in Response #24. However, to improve readability, we have separated the data into two figures (Figure 2 and Figure 3), distinguishing between Sherman and Wire mesh traps.*

Table 3 – Appears to be missing.

*Response #27:* *Indeed, Table 3 was inaccurately labled as Table 4. This was corrected in the revised version of our manuscript. (see line Table 3)*

**Table 4 –** This table is incomprehensible as currently displayed. What do the column headings mean? What do the values mean? What are the stats related to and what are they? What is the message of this table?

*Response #28: Thank you for your valuable feedback. We acknowledge that the previous table layout may have made it difficult for readers to assess trapping effort and the robustness of zero values. To improve clarity, we have modified the presentation of the table to include all neighborhoods for all species, making it easier to identify suspected true absences (i.e., no capture despite strong trapping effort) and low trapping effort that may also explain absence of capture. We believe this adjustment now ensures a more transparent presentation of the results, and simplifies their interpretation for readers.*

*Additionally, we have decided not to include statistical values directly in the table to avoid unnecessary complexity. However, we now propose to have the initial table, including the statistical values, as a supplementary material. All statistical test results are also carefully reported in the text, ensuring that this important information remains easily accessible to readers.*

*All abbreviations are now included in the revised legend, which now reads as follows: “TN” and “N” stand for the number of trap-nights (i.e., the total number of nights during which traps were set, representing the trapping effort), "N" refers to the number of captures, and "CR" represents the capture rate (i.e., the ratio of captures to trap-nights, used as a measure of trapping success or yield, see lines 244-248).*

Line 225 – This is misleading as not only did Mus musculus not “appear to expand massively” between P1 and P2, it did not expand at all if the number of positive sites is the metric used.

*Response #29: We agree and confirm that Mus musculus did not expand between the two periods (P1 and P2), with the same single positive site during P1 campaign remaining unchanged during P2 one. From there, our statement was indeed unclear. Accordingly, it has been reworded in the revised version of the manuscript, and it now reads: “**When one considers captures obtained with both Sh and Wm traps, that M. natalensis was found in eight localities during both periods P1 and P2” (Line 205-206).*

Line 236 – Please clarify what is meant by “our observational device”.

*Response #30: The term 'our observational device' refers to the set of localities that were surveyed during both periods.*

Line 237 – ‘Support’ should be used in place of ‘confirm’.

*Response #31:* *This has been modified accordingly (see line 284)*

Lines 238-239 – How would an efficient vs. inefficient colonization be distinguished? What characteristics would be observed if Rattus rattus was not showing a ‘quite efficient colonization’ of the city vs. what was observed here?

*Response #32: We agree that the term “efficient” is not appropriate. So, in the text now by “successful” (see line 286).*

Line 243-255 – What about features of rodent biology might make this neighborhood make it more suitable to M. natalensis over Rattus rattus?

*Response #33: The characteristics that elucidate the disparate dynamics between Rattus and Mastomys natalensis remain to be elucidated and have yet to be the focus of in-depth study in the context of Niamey. However, the existing body of literature has identified potential drivers of these dynamics, including eco-evolutionary processes such as competition for resources, parasite sharing, and environment-related reproduction rates, as well as socio-ecosystemic characteristics like human actions against parasites and changes in food supply. These elements have been incorporated into the manuscript (lines 302-306, which reads " However, the eco-evolutionary processes (e.g., competition for resources, parasite sharing, environment-mediated reproductive rate) and/or socio-ecosystem characteristics (e.g., human action against pest, human food provisioning) that explain such different dynamics of Rattus rattus – Mastomys natalensis remain unknown and still to be investigated in details.").*

**The discussion** about how R. rattus is spreading in the city is too light on alternative explanations other than the type of building/building material. Are there other features of the way the city is connected/used that might explain this? There is extensive literature regarding rodent invasion in other cities that should be referred to here.

*Response #34: Thank you for suggesting us to explore additional references regarding rodent invasion in cities. More explanations and related discussion items have been added in the discussion section of the manuscript.*

Lines 259-260 – The claim that Rattus rattus presence/abundance is negatively associated with M. natalensis should be statistically tested. Please include in the analysis.

*Response #35: After careful examination, we agree that statistical test the correlation between the increase in Rattus and the decrease in Mastomys has not been tested in the paper. Now it is done using two different methods whose were both congruent and highly significant (r=-0.671; p=0.001, see S1 Fig for more details).*

Line 272-273 – Seoul virus is properly called Orthohantavirus seoulense (ICTV) and should be italicized. Otherwise suggest referring to only the common name of Seoul virus as Seoul virus, as what is currently written is neither correct taxonomy or accepted common name.

*Response #36: We have corrected this by using the proper name Orthohantavirus seoulense (italicized) or simply "Seoul virus" when referring to the common name (see line 326).*

Line 275 – Similarly, Lassa Mammarenavirus should be referred to as Mammarenavirus lassaense (ICTV) or Lassa virus.

*Response #37: We have made the correction and now use Mammarenavirus lassaense (italicized) or "Lassa virus" when referring to the common name (see line 329).*

Lines 281-283 – Please include references to support this statement.

*Response #38: References have been added to support this statement (see lines 337).*
