# Supplementary material for: Native-to-invasive rodent species turn-over within African cities: The example of Niamey, Niger
Source: PLoS One. 2025 Jul 22;20(7):e0325427. doi: 10.1371/journal.pone.0325427 (PMC12282928; doi:10.1371/journal.pone.0325427)
Supplement: Attachment — Submitted filename: Response_to_Reviewers_auresp_2.docx [file pone.0325427.s006.docx]

Université Abdou Moumouni de Niamey

Faculté des Sciences et Techniques

Département de Biologie

BP. 10662, Niamey, NIGER

Tel: 227 + 98136998

Email: [az.danzabarma@gmail.com](mailto:az.danzabarma@gmail.com)

*Niamey, Friday 9^nd^ May 2025*

Dear *Plos One* Editorial Board,

Please find attached our re-revised manuscript titled “Native-to-invasive rodent species turn over within African cities: an example in Niamey, Niger” by Abdoulaziz IBRAHIM DANZABARMA and colleagues. We would like to sincerely thank the editorial board for their constructive comments.

In response to the editor’s comments and the Journal Requirements, we have made corrections mainly related to grammar and vocabular (singular/plural agreements), word omissions, and rewording certain sentences for better understanding. Concerning the comments on the reference list, we would like to precise that there is no new references added or removed after revising the manuscript. The references mentioned at line **360**, as requested by the reviewer #3, were already cited in the text. It concerns, references [32-37], which have simply been reintroduced to support the idea discussed in the text.”

Please find below our detailed point-by-point responses to the others comments. We hope that these revisions meet the journal's expectations and that our manuscript will be accepted for publication.

Kind regards with thanks and all the best wishes

On behalf of the manuscript’s authors

Abdoulaziz IBRAHIM DANZABARMA

**Manuscript #** **PONE-D-24-55809: Native-to-invasive rodent species turn-over within African cities: the example of Niamey, Niger**

COMMENTS FROM ACADEMIC EDITOR: Dr Javier delBarco-Trillo

Academic Editor

Comments to the Author:

Dear Dr. Ibrahim Danzabarma,

Thank you for submitting your manuscript to PLOS ONE. After careful consideration, we feel that it has merit but does not fully meet PLOS ONE’s publication criteria as it currently stands. Therefore, we invite you to submit a revised version of the manuscript that addresses the points raised during the review process.

Please submit your revised manuscript by Jun 06 2025 11:59PM. If you will need more time than this to complete your revisions, please reply to this message or contact the journal office at [plosone@plos.org](mailto:plosone@plos.org). When you're ready to submit your revision, log on to <https://www.editorialmanager.com/pone/> and select the 'Submissions Needing Revision' folder to locate your manuscript file.

Please include the following items when submitting your revised manuscript:

- A rebuttal letter that responds to each point raised by the academic editor and reviewer(s). You should upload this letter as a separate file labeled 'Response to Reviewers'.
- A marked-up copy of your manuscript that highlights changes made to the original version. You should upload this as a separate file labeled 'Revised Manuscript with Track Changes'.
- An unmarked version of your revised paper without tracked changes. You should upload this as a separate file labeled 'Manuscript'.

If you would like to make changes to your financial disclosure, please include your updated statement in your cover letter. Guidelines for resubmitting your figure files are available below the reviewer comments at the end of this letter.

If applicable, we recommend that you deposit your laboratory protocols in [protocols.io](http://protocols.io/) to enhance the reproducibility of your results. Protocols.io assigns your protocol its own identifier (DOI) so that it can be cited independently in the future. For instructions see: [https://journals.plos.org/plosone/s/submission-guidelines#loc-laboratory-protocols](https://track.editorialmanager.com/CL0/https:%2F%2Fjournals.plos.org%2Fplosone%2Fs%2Fsubmission-guidelines%23loc-laboratory-protocols/1/010f01965f1ecc1e-fe3e67d7-00a7-4a64-8e24-5fc79bda16c2-000000/wLZaDfcuDLXZPsZYCPO-JnD1LxON10izMKGk5pB3X2g=208). Additionally, PLOS ONE offers an option for publishing peer-reviewed Lab Protocol articles, which describe protocols hosted on [protocols.io](http://protocols.io/). Read more information on sharing protocols at [https://plos.org/protocols?utm_medium=editorial-email&utm_source=authorletters&utm_campaign=protocols](https://track.editorialmanager.com/CL0/https:%2F%2Fplos.org%2Fprotocols%3Futm_medium=editorial-email%26utm_source=authorletters%26utm_campaign=protocols/1/010f01965f1ecc1e-fe3e67d7-00a7-4a64-8e24-5fc79bda16c2-000000/8iCHxTnHQSX6I2JZoEPDE9e0vz_VvpAicvGmd-ZcuVQ=208).

We look forward to receiving your revised manuscript.

Kind regards,

Javier delBarco-Trillo, Ph.D.

Academic Editor

PLOS ONE

Journal Requirements:

Please review your reference list to ensure that it is complete and correct. If you have cited papers that have been retracted, please include the rationale for doing so in the manuscript text, or remove these references and replace them with relevant current references. Any changes to the reference list should be mentioned in the rebuttal letter that accompanies your revised manuscript. If you need to cite a retracted article, indicate the article’s retracted status in the References list and also include a citation and full reference for the retraction notice.

Additional Editor Comments:

The authors have addressed most of the reviewers’ comments satisfactorily, so I will not send the manuscript for another round of reviews. However, I have identified several issues that still need to be addressed, some of them involving the new text in the revision.

For specific comments referring to the text, the listed lines below refer to the version with Track changes.

***Response #1:*** *We sincerely thank the Editor for his constructive comments on the revised version of our manuscript. We appreciate very much the time and attention devoted to improving our work.*

*We are pleased that the revised version of our manuscript has addressed satisfactorily most of the reviewers' comments. We would like also to thank you for the opportunity you give us to submit a further revised version. According to the editor’s comments, we have thoroughly reviewed the entire manuscript and made the necessary changes, including the newly added sections and the list of references as requested. All modifications and corrections made are available in the re-revised version of the manuscript named “Revised Manuscript with Track Changes.docx”.*

*Please find below both detailed response to each comment and precise indication of the changes made in the revised manuscript, referring to the line numbers in the annotated version.*

*We hope that these revisions fully address the latest editor’s concerns.*

If you need to cite a retracted article, indicate the article’s retracted status in the References list and also include a citation and full reference for the retraction notice

Consider if some of the methodological information you offered in responses #19 and #21 shouldn’t be incorporated into the Methods section. You make good points, and such points being stated in the Introduction may minimize similar questions from other readers.

***Response #2:*** *We agree that this observation is relevant to readers. It was therefore retained and the information was added in the material and methods section. the Response 21 is in* ***lines 103*** ***to 10-*** *and reads (Additional data from extra localities were collected across Niamey during the P2 period, thus allowing us to document further the extant distributions of native and invasive species observed to date in Niamey. That may be of interest for other purposes, including eco-evolutionary meta-analyses, niche modeling, zoonotic pathogen screenings, etc.).*

*The elements of answer 19 are found* ***on lines 147 to 155*** *(To assess long-term changes in urban rodent communities over a ten-year period, we compared trapping data collected during two distinct campaigns: P1 (2009-2013) and P2 (2020-2023). The P1 campaign spanned two years, while P2 extended over three years. Given the logistical constraints associated with large-scale urban sampling (i.e., 987 individuals from 52 intra-Niamey neighborhoods during P1 [16], and 604 captures from 21 neighborhoods during P2 [this study]), it was not feasible to sample all localities within the same months or seasons during the same campaigns. As a result, trapping sessions occurred at different times of the year. However, due to the absence of significant seasonal migration patterns in urban rodent populations, data collected across different seasons within each campaign could be pooled.)*

Line 39, as there are two, it should be “genera” and not “genus”. But I would refer to the cosmopolitan species, basically only three (brown and black rat and house mouse), and not to the genera, as between these two genera you have over 100 species and most of them are not cosmopolitan.

***Response #3:*** *Thank you for this comment. We fully agree and now refer only to the three cosmopolitan species—Rattus norvegicus, Rattus rattus, and Mus musculus—instead of the genera Rattus and Mus, which indeed include many species. The associated change can be found* ***on line 41****: “…* such as *Rattus norvegicus, Rattus rattus* and *Mus musculus …*”*].*

Line 81, correct “These sites households”

***Response #4:*** *The sentence was indeed incomplete and has been corrected as: "These sites are households…" (****line 83****).*

Line 85, correct “with an effort of sampling 2 to 4 sites per locality”

***Response #5:*** *The sentence has now been revised to "*Between 4-18 sites were sampled in each locality*"* ***(line 89-89****).*

Line 98: “space”. Same singular/plural inconsistency in line 103: it should be “a new baited trap”

***Response #6:*** *We corrected the singular/plural inconsistency by removing the "s" from both "spaces" and "traps" (****on lines 107 and 111, respectively****).*

Lines 144-148: “yellow points indicate localities sampled at different sites across periods, and green points show new localities sampled in Period 2” is a bit confusing. Do you mean that “yellow points indicate localities sampled in Period 1 that were not resampled in Period 2”?

***Response #7:*** *Yes, that’s close to what we mean. To make it clearer, the legend has been changed to “**The green dots indicate localities sampled only in P2.* *Localities sampled in both P1 and P2 are represented on red if sample sites are the same between the two periods and on yellow if the sites are different.”* ***Line 162-165.***

Table 2. Check the title row, there is a problem with it.

***Response #8:*** *Indeed,* *there was a mismatch between columns. This has now been corrected* ***(see Table 2).***

Line 202, to address reviewer 3’s comment, I would consider changing “significant” for “substantial”, “high”, “strong” or a similar term that denotes the meaning intended while avoiding any confusion with the statistical usage of the term “significant”.

***Response #9:*** *Accordingly, the term “high” now replaces “significant”, thus better reflecting the intended meaning of this sentence. (****see line 221****).*

Line 205, something is missing in “both Sh and Wm traps, it appears that M. natalensis”

***Response #10:*** *The sentence now reads: “*When one considers captures obtained with both Sh and Wm traps, *M. natalensis* was found in eight localities during both periods P1 and P2*.” (****see line 224****).*

Lines 246-249, please check the quotations and make sure they are being used appropriately and consistently. I think in line 228 you want to write “TN” stand for the…”, as “N” is defined later.

***Response #11:*** *Indeed, the correct intended abbreviation should be “TN,” not “NT.” This has now been corrected. Note that the use of citations was also carefully checked again throughout the text to ensure consistency and appropriateness.*

Please correct small typos. For example, in line 275 remove the period after “Table”
***Response #12:*** *This has been done accordingly.* ***See line or table 297***

In many places, shouldn’t it be “Table S1” instead of “S1 Table”?

***Response #13:*** *The journal's template, specifies that supporting information should be cited as "S1 Table," "S1 Fig,". So, we used this format consistently throughout the manuscript to ensure compliance with the journal's guidelines. However, we found that in all the articles published by PLoS One that we have read, the supplementary annotation is* “Table S1” and “Figure S1”. As a consequence, we finally considered this latter format (Table S1, Fig S1, etc.) for own manuscript, hoping it will reach expected editorial standards.

Live 284: I agree with Reviewer 3 that “observational device” is a confusing term even if correct, as many readers will primarily associate “device” with a physical object. Please use other term, e.g. “strategy” or “approach”. Or maybe you don’t need “of our observational device” at all?

***Response #14:*** *We concede that this may be a confusing term so, as suggested, it was completely removed from the text (****see line 306****).*

Line 287, avoid using “quite” to add uncertainty

***Response #15:*** *Accordingly, the term “quite” has now been removed to make the statement more precise and confident.* ***(see line 309)***

Line 307, instead of “unknown and still to be investigated in details” you can use “to be investigated”

***Response #16:*** *This has been* *modified accordingly (****see line 329****).*

Line 312, “Fig. S1”

***Response #17: “****S1 Fig”. has been corrected into “Fig. S1” (****see line 334****).*

Lines 327 and 330, most readers won’t know what ICTV stand for, and probably will appreciate the common name.

***Response #18:*** *Thank you for your comment. The common names of Lassa virus and Seoul virus have now been written in full length (****see lines 348-349 and 351-352****).*

Line 359, “(DOC)”?

***Response #19:*** *The term "DOC" in the caption was added to indicate the file extension. Maybe it is not our responsibility to include it, so it has now been removed from the manuscript. (****see line 381****).*

Lines 355 and 360, why “Table.” and “Fig:”?

***Response #20:*** *We used “Table” in full and “Fig.” in abbreviated form because this is how they appear in the journal’s Supporting Information template (e.g., “S1 Fig.”, “S1 Table.”). However, in this last version of our manuscript, the considered format now follows that described in Response #13 (i.e., Table S1, Fig. S1, etc.).*

Line 360, again, please go through manuscript and pay attention to small details. Here, do not italicize the “and” between species names

***Response #21:*** *This has now been modified and checked through all the manuscript. (****see line 382****).*

Line 362, “The red dots are for negative ratios and the blue dots for positive ratios”

***Response #22:*** *Following my response #25, we revisited the figure by assigning the color code to the period. The red dots for the ratios of period 1 and the blues, those of period 2.*

Line 363: “Spearman”

***Response #23:*** *Accordingly,* *the lowercase “s” in “spearman” has been modified into an uppercase “S”. (****see line 387****)*

Fig S1 legend, you use both “figure” and “frame” to refer to the panels. Use “panel” instead

***Response #24:*** This was modified accordingly.

Fig S1, I find the left panel very confusing. You say you want to portray the change between the two periods, but there is no clear indication about the two periods. The reader will think there is something important about the two colors, but they simply identify points over or under 0, but that’s quite confusing. You could have a solid line at 0, which would easily show points above and under. As for the right panel, please use common notation with “r = -.0671, p < 0.01” or whatever the values are.

***Response #25:*** *As kindly suggested, the issue about the left panel was addressed through the addition of a solid line at 0, which now clearly separates positive and negative, thus helping to visualize changes between the two periods. Additionally, the colors of the points have been assigned to the period to avoid confusion. Thus, the reader will be able to read that the red points correspond to the ratios in period 1 and the blues, the ratios in period 2.*

*In the right panel, the notation is “r = -0.671, p < 0.01,” was added, as suggested, too.*

Table S1, you are providing p values, so what’s the point of adding asterisks or ns? That’s redundant.

***Response #26:*** *Accordingly, since p-values are provided, asterisks or "ns" were indeed redundant and has now been removed.*

References: please check for consistency across references. For example, in many references you include the doi (correctly), whereas in others you add a website (even though a doi is available).

***Response #27:*** *We are sorry about this lack of consistency: references were all checked and reviewed, accordingly, and doi should now be correctly provided.*

[Note: HTML markup is below. Please do not edit.]

[NOTE: If reviewer comments were submitted as an attachment file, they will be attached to this email and accessible via the submission site. Please log into your account, locate the manuscript record, and check for the action link "View Attachments". If this link does not appear, there are no attachment files.]

While revising your submission, please upload your figure files to the Preflight Analysis and Conversion Engine (PACE) digital diagnostic tool, [https://pacev2.apexcovantage.com/](https://track.editorialmanager.com/CL0/https:%2F%2Fpacev2.apexcovantage.com%2F/1/010f01965f1ecc1e-fe3e67d7-00a7-4a64-8e24-5fc79bda16c2-000000/dD83eDC0y1X2HZ2mxwapNkSgTcdrMxDhniw5njQFmAg=208). PACE helps ensure that figures meet PLOS requirements. To use PACE, you must first register as a user. Registration is free. Then, login and navigate to the UPLOAD tab, where you will find detailed instructions on how to use the tool. If you encounter any issues or have any questions when using PACE, please email PLOS at [figures@plos.org](mailto:figures@plos.org). Please note that Supporting Information files do not need this step.
